# Candidate chemosensory genes identified from the greater wax moth, *Galleria mellonella*, through a transcriptomic analysis

**DOI:** 10.1038/s41598-019-46532-x

**Published:** 2019-07-11

**Authors:** Hong-Xia Zhao, Wan-Yu Xiao, Cong-Hui Ji, Qin Ren, Xiao-Shan Xia, Xue-Feng Zhang, Wen-Zhong Huang

**Affiliations:** 1 Guangdong Key Laboratory of Animal Conservation and Resource Utilization, Guangdong Public Laboratory of Wild Animal Conservation and Utilization, Guangdong Institute of Applied Biological Resources, Guangzhou, 510260 PR China; 2 Guangzhou Academy of Agricultural Sciences, Guangzhou, 510308 PR China; 3 Chongqing Academy of Animal Science, Chongqing, 402460 PR China

**Keywords:** Gene expression, Transcriptomics

## Abstract

The greater wax moth, *Galleria mellonella* Linnaeus (Lepidoptera: Galleriinae), is a ubiquitous pest of the honeybee, and poses a serious threat to the global honeybee industry. *G. mellonella* pheromone system is unusual compared to other lepidopterans and provides a unique olfactory model for pheromone perception. To better understand the olfactory mechanisms in *G. mellonella*, we conducted a transcriptomic analysis on the antennae of both male and female adults of *G. mellonella* using high-throughput sequencing and annotated gene families potentially involved in chemoreception. We annotated 46 unigenes coding for odorant receptors, 25 for ionotropic receptors, two for sensory neuron membrane proteins, 22 for odorant binding proteins and 20 for chemosensory proteins. Expressed primarily in antennae were all the 46 odorant receptor unigenes, nine of the 14 ionotropic receptor unigenes, and two of the 22 unigenes coding for odorant binding proteins, suggesting their putative roles in olfaction. The expression of some of the identified unigenes were sex-specific, suggesting that they may have important functions in the reproductive behavior of the insect. Identification of the candidate unigenes and initial analyses on their expression profiles should facilitate functional studies in the future on chemoreception mechanisms in this species and related lepidopteran moths.

## Introduction

Olfaction is essential for the survival and reproduction of insects. Antennae are crucial olfactory appendages in the olfactory system, which perceives various chemical stimuli typically via two steps. First, odorant molecules penetrate the sensillar lymph through cuticular pores, where they are recognized by soluble olfactory proteins, i.e. odorant-binding proteins (OBPs)^1,2^ or potential chemosensory proteins (CSPs)^3^. Second, the ligand-bound OBPs or CSPs transfer the odorant molecules across the lymph to olfactory receptors in the dendritic membrane of olfactory sensory neurons (OSNs), which then activate and generate signals that trigger the insect’s response to the stimulus^2,4,5^. Olfactory receptors include odorant receptors (ORs) and ionotropic receptors (IRs). These olfactory gene families have been proved as suitable targets for designing semiochemicals for pest control^6–10^. In addition, sensory neuron membrane proteins (SNMPs), which are located on dendrites of OSNs, are proposed to be associated with pheromone reception^11–13^.

The greater wax moth, *Galleria mellonella* Linnaeus (Lepidoptera: Galleriinae), is a ubiquitous pest of honeybee colonies. *G. mellonella* larvae destroy honeycomb and feed on wax, bee larvae, pollen and honey. This pest poses a serious threat to the global honeybee industry^14^. Control measures for this pest include heat treatments, chemical fumigation, and the male sterilization. However, each of these measures has unresolved downsides^14^. Heat treatments are only applicable in the absence of living honeybee stages and in small-scale beekeeping because unaffordable costs would be required for large-scale bee farms. Chemical fumigation poses health risks to handlers and may lead to toxic residues in bee products such as honey. Male sterilization requires a high input cost and could lead to exacerbate economic losses. Therefore, a better understanding on the biology of this species is needed to search for novel methods to control this pest.

A better understanding of components and pathways that are involved in olfactory communication could lead to effective methods for pest control. In the process of mating, most of lepidopterans, the female produce pheromone blends that stimulates male receptivity toward females, and only males detect female pheromones with specialized neurons on their antennae that express male-specific pheromone receptors^2,15^. However, in *G. mellonella*, the male produced acoustics courtship behaviour that attracts virgin females to males. Once in close proximity, the male releases sex pheromone blends including nonanal, decanal, hexanal, heptanal, undecanal, and 6, 10, 14 trimethylpentacanol-2 and 5, 11-dimethylpentacosane^16–20^, which serve as short-range chemical cues that initiates mating. This is particularly true since *G. mellonella* displays a unique olfactory system of sex pheromone perception, and little is known about the genes and molecular events involved in chemoreception. Investigation on the molecular components of the olfactory organ in *G. mellonella* should provide insights into olfactory mechanisms for pheromone perception in *G. mellonella*, and could ultimately lead to the development of behavior-based pest control strategies. In this study, we generated antennal transcriptomes of *G. mellonella* using high-throughput sequencing, and annotated gene families encoding putative ORs, IRs, SNMPs, OBPs and CSPs. Expression profiles of these unigenes in different tissues were also investigated using semi-quantitative RT-PCR and real-time quantitative-PCR. In addition, evolutionary relationships of the identified unigenes with olfaction genes from other lepidopterans were also analyzed. These results should provide a foundation for future functional characterization of the olfactory genes in *G. mellonella*.

## Results

### Transcriptome sequencing

To identify candidate chemosensory genes, transcriptome was generated from antennae dissected from both males and females, separately, using Illumina HiSeq 4000 platform. After removing low quality reads, adaptor, and contaminating sequence reads, approximately 41.5 million and 36.8 million clean reads were generated from female and male antennae, respectively. Trinity assembly of the clean reads from both male and female antennae resulted in 65,593 transcripts with a mean length of 1,388 bp and an N50 length of 2,511 bp. 54,234 unigenes were selected from the above transcripts with a mean length of 1,125 bp and an N50 length of 2,131 bp (Table S1).

### Putative odorant receptors

Form the assembled unigenes, 46 of them were annotated encoding ORs, which were found to belong to the 7-transmembrane receptors superfamily. Of these OR unigenes, 34 represented full-length ORFs encoding more than 355 amino acid residues, and with 5–10 transmembrane domains (TMDs), which are characteristic of typical insect ORs.

**Figure 1:**
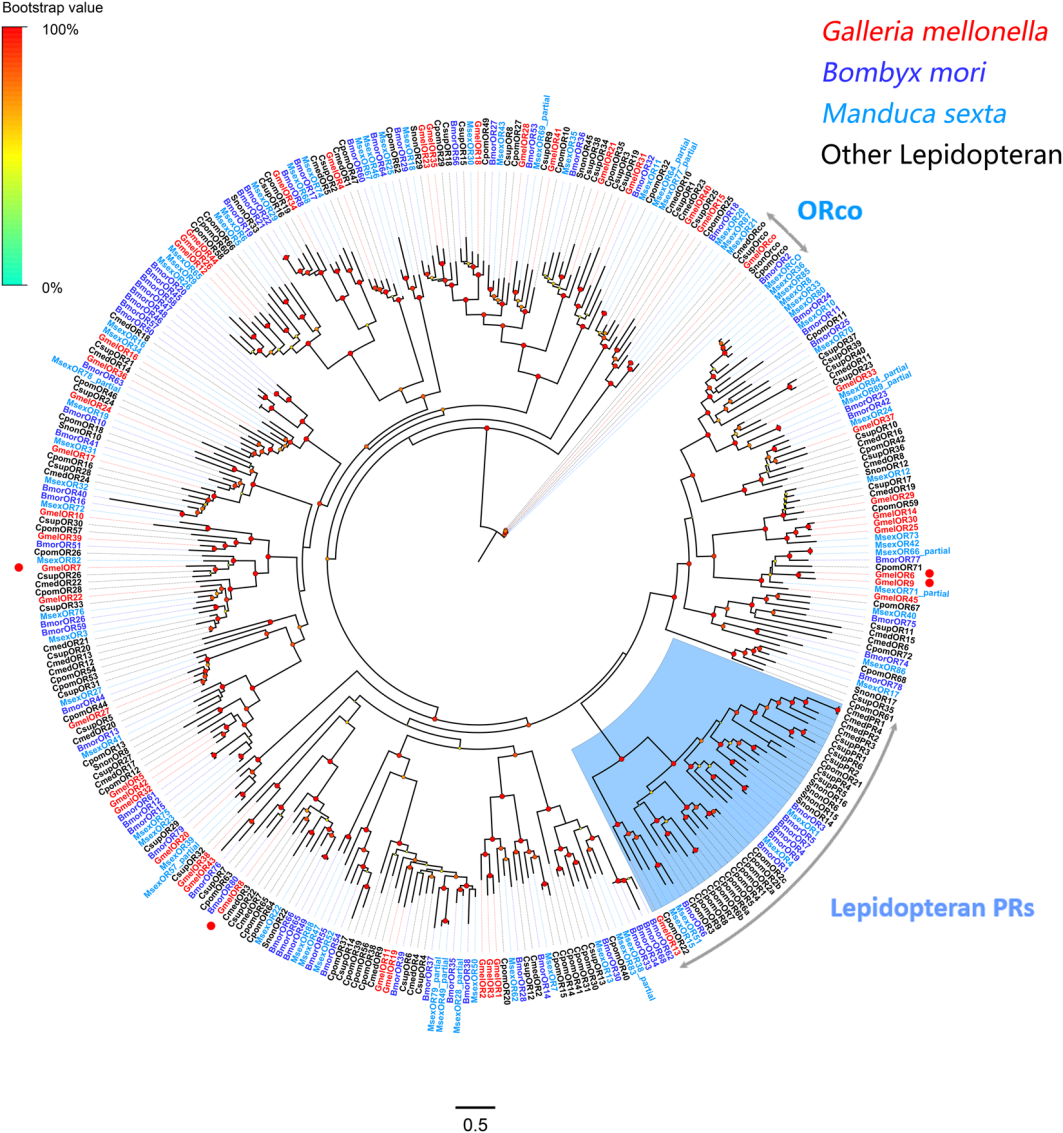
Maximum-likelihood tree of ORs. The distance tree was rooted by the conservative Orco gene orthologues. Species abbreviations: Gmel, *Galleria mellonella*; Bmor, *Bombyx mori*; Cpom, *Cydia pomonella*; Csup, *Chilo suppressalis*; Msex, *Manduca sexta*; Snon, *Sesamia nonagrioides*. Highlighted clades: the lepidopteran pheromone receptor (PR) clade in blue. Node support (circles at the branch nodes) was estimated using an approximate likelihood ratio test based on the scale indicated at the top left. Bars indicate branch lengths in proportion to amino acid substitutions per site.

A phylogenetic analysis with the 46*G. mellonella* ORs together with ORs from other lepidopterans including *Bombyx mori*^21^, *Cnaphalocrocis medinalis*^22^, *Cydia pomonella*^23^, *Chilo suppressalis*^24^, *Manduca sexta*^25^ and *Sesamia nonagrioides*^26^ revealed that the *G. mellonella* OR co-receptor named GmelOrco formed a clade with the protein Orcos from other lepidopterans (Fig. 1). The other *G. mellonella* ORs were assigned to different clades with other known lepidopteran ORs. However, no OR from *G. mellonella* belonged to the lepidopteran PR clade. Several species-specific branch was detected including GmelOR1/2/3, GmelOR14/25/30, GmelOR5/32/42 and GmelOR12/26/44. Information including unigene sequences, lengths, best blastx hits and predicted protein domains of all 46 putative ORs are listed in the Supplementary Dataset: Sheet 1.

### Putative ionotropic receptors

Twenty-five putative iGluRs/IRs were identified from the *G. mellonella* antennal transcriptomes. Of these iGluRs/IRs, 18 had full-length ORFs encoding proteins with at least 547 amino acid residues. All these full length candidate unigenes were predicted to encode ligand-gated cation channels (S1 and S2) with three transmembrane domains (M1, M2, and M3), or portions of the domains (Supplementary Dataset: Sheet 2 and Fig. S1), which was consistent with the characteristics of insect iGluRs/IRs.

**Figure 2:**
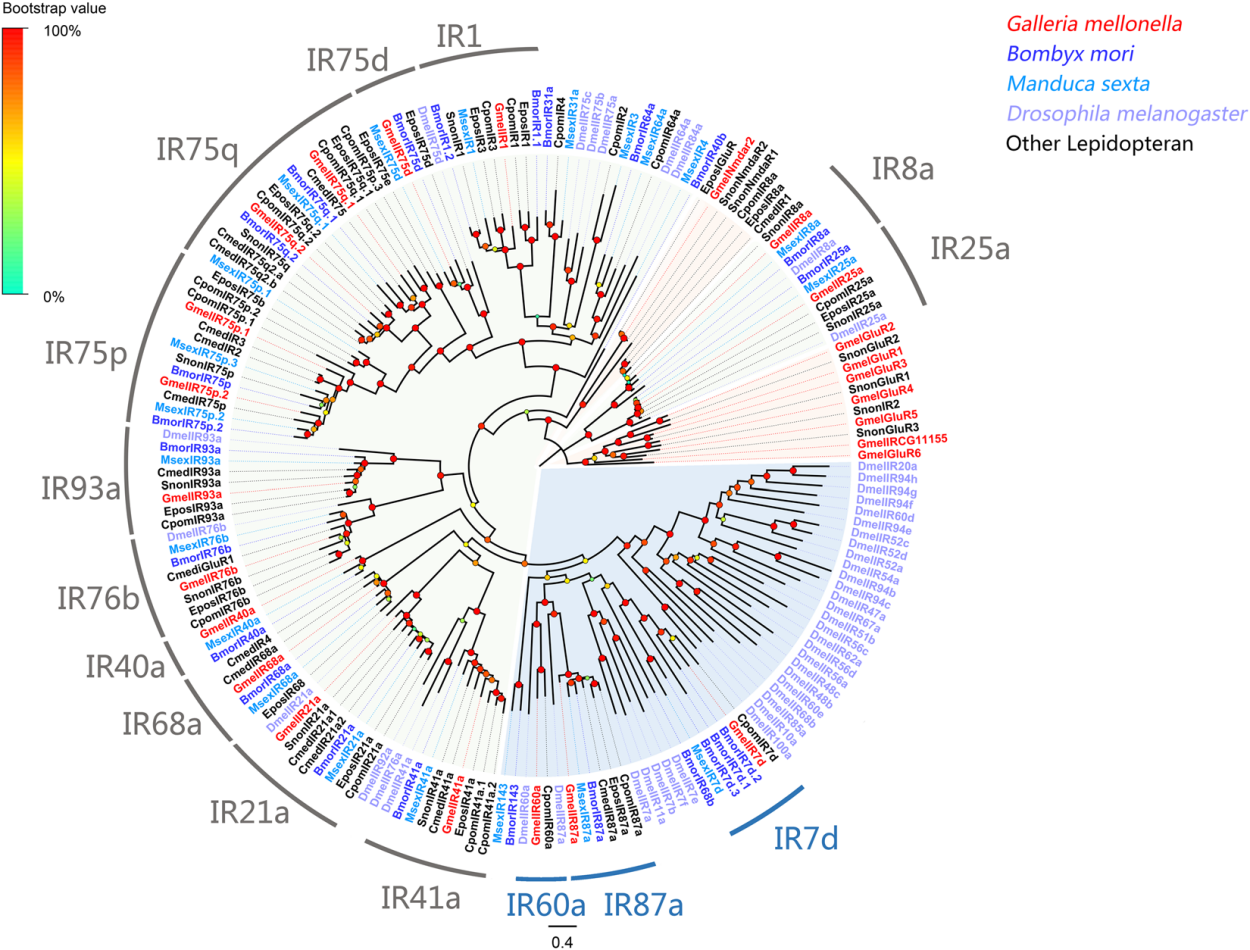
Maximum-likelihood tree of iGluRs/IRs. The distance tree was rooted by the conservative IR25a/IR8a gene orthologous. Species abbreviations: Gmel, *Galleria mellonella*; Bmor, *Bombyx mori*; Cmed, *Cnaphalocrocis medinalis*; Cpom, *Cydia pomonella*; Epos, *Epiphyas postvittana*; Msex, *Manduca sexta*; Snon, *Sesamia nonagrioides*; Dmel, *Drosophila melanogaster*. Highlighted clades: non-N-Methyl-D-aspartic acid (purple), iGluRs (orange), antennal IRs (green) and divergent IRs (blue). Node support (circles at the branch nodes) was estimated using an approximate likelihood ratio test based on the scale indicated at the top left. Bars indicate branch lengths in proportion to amino acid substitutions per site.

A phylogenetic analysis was conducted using the identified putative *G. mellonella* iGluRs/IRs with iGluRs/IRs from other lepidopterans including *B. mori*^27^, *C. medinalis*^22^, *C. pomonella*^23^, *C. suppressalis*^24^, *M. sexta*^25^, *S. nonagrioides*^26^ and *Drosophila melanogaster*^27^. All identified *G. mellonella* iGluRs/IRs were clustered with their orthologs from *D. melanogaster* and other lepidopteran species, and were assigned to four phylogenetic groups including non-N-Methyl-D-aspartic acid (NMDA), iGluRs, antennal IRs and divergent IRs (Fig. 2). Information including unigene sequences, lengths, best blastx hits and predicted protein domains of the identified IRs are listed in Supplementary Dataset: Sheet 2.

### Putative sensory neuron membrane proteins

**Figure 3:**
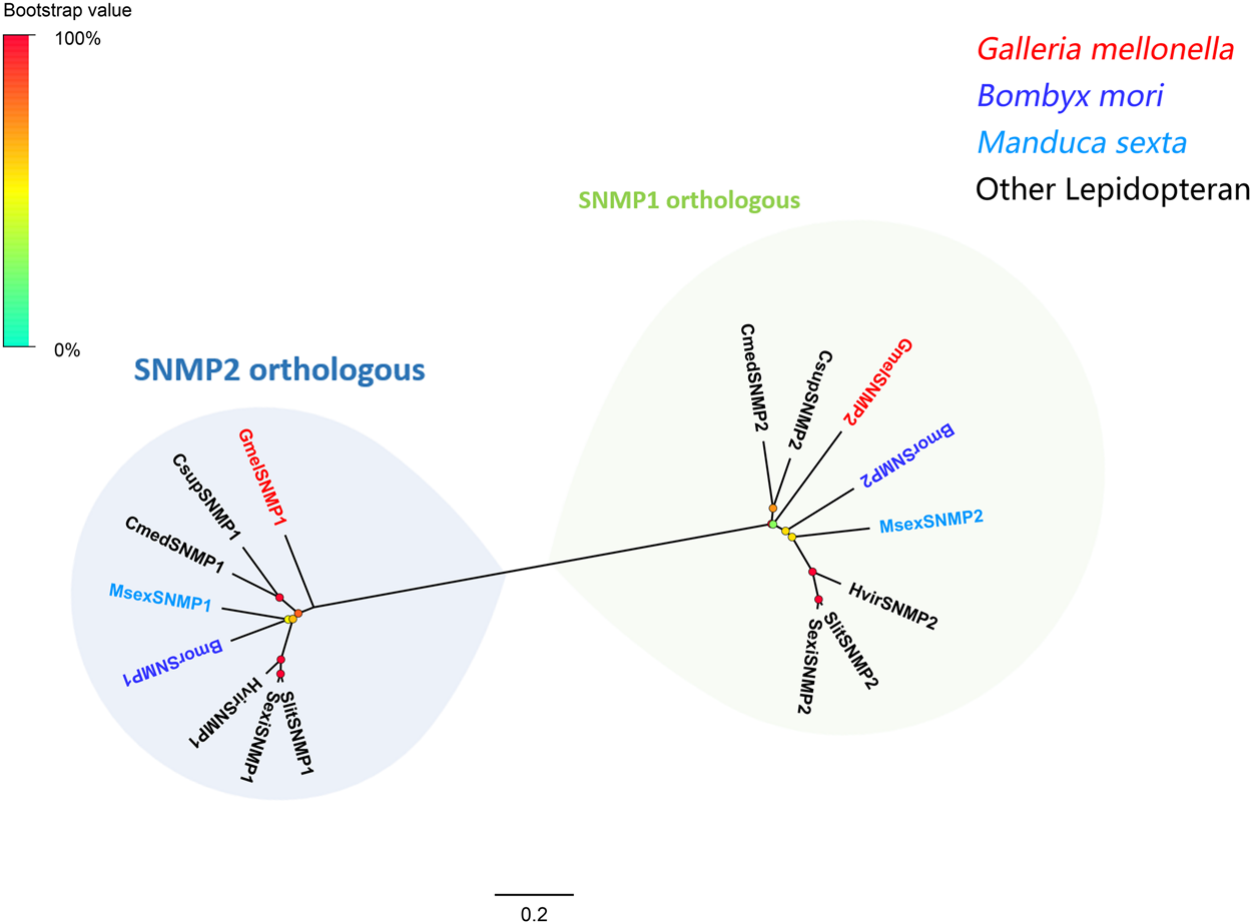
Maximum-likelihood tree of SNMPs. Species abbreviations: Gmel, *Galleria mellonella*; Bmor, *Bombyx mori*; Cmed, *Cnaphalocrocis medinalis*; Msex, *Manduca sexta*; Snon, *Sesamia nonagrioides*; Slit, *Spodoptera littoralis*; Hvir, *Heliothis virescens*. Highlighted clades: SNMP1 clade (green) and SNMP2 clade (blue). Node support (circles at the branch nodes) was estimated using an approximate likelihood ratio test based on the scale indicated at the top left. Bars indicate branch lengths in proportion to amino acid substitutions per site.

Two SNMPs, named GmelSNMP1 and GmelSNMP2, were annotated from the *G. mellonella* transcriptomes. Both SNMPs are full length with ORFs encoding proteins with at least 540 amino acid residues. Phylogenetic analyses were carried out on the *G. mellonella* SNMPs together with SNMPs from other insects including *B. mori*^12^, *C. medinalis*^22^, *M. sexta*^12^, *S. nonagrioides*^26^, *Spodoptera littoralis*^28^, *Heliothis virescens*^12^ and *D. melanogaster*^12^. As expected, GmelSNMP1 and GmelSNMP2 were clustered with SNMP1 and SNMP2 orthologues from other lepidopterans, respectively (Fig. 3). Information including unigene sequences, lengths, best blastx hits and predicted protein domains of the two SNMPs are listed in Supplementary Dataset: Sheet 3.

### Putative odorant binding proteins

**Figure 4:**
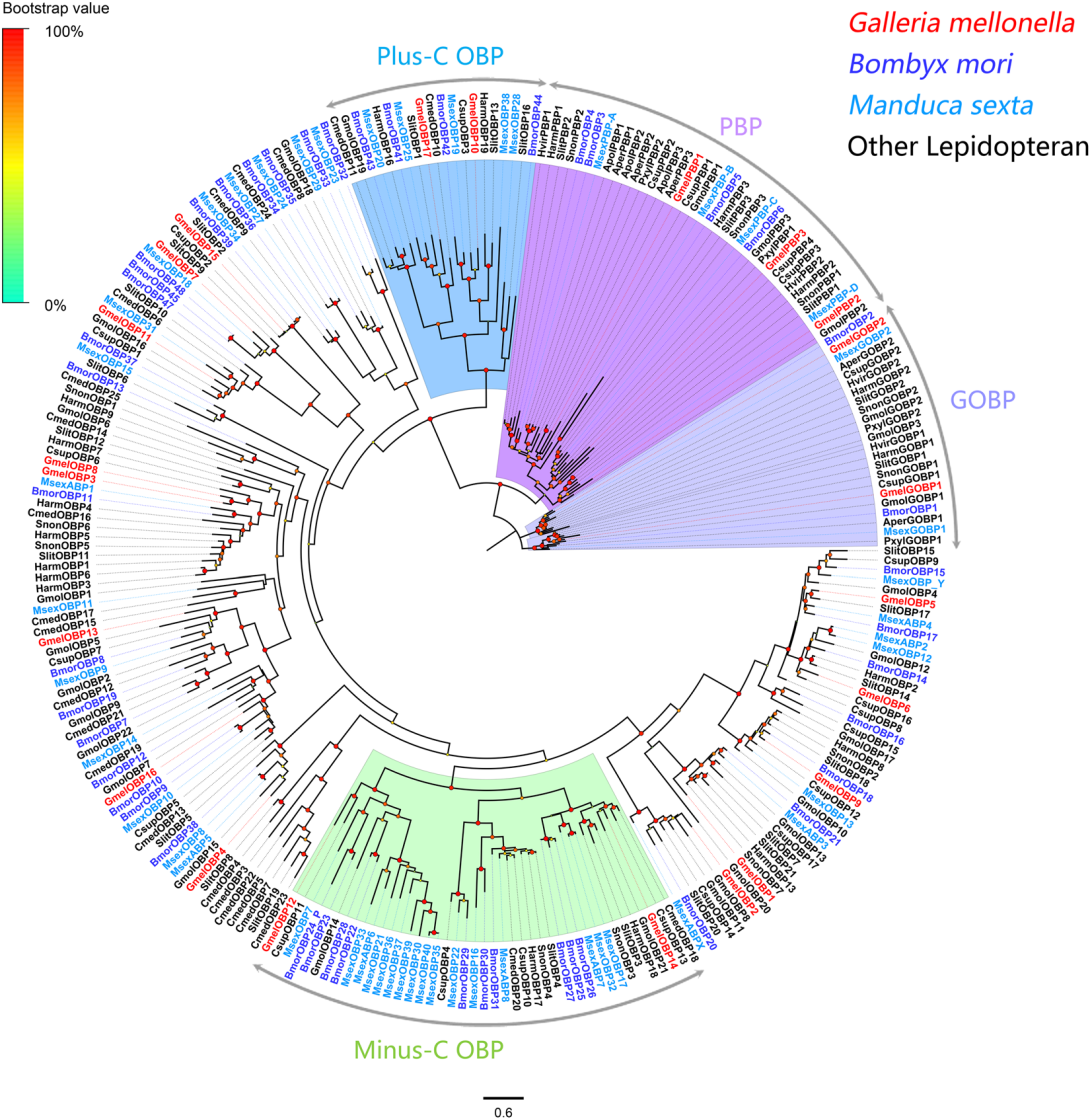
Maximum-likelihood tree of OBPs. The distance tree was rooted by the conservative GOBP and PBP gene orthologous. Species abbreviations: Gmel, *Galleria mellonella*; Apol, *Antheraea Polyphemus*; Aper, *Antheraea pernyi*; Bmor, *Bombyx mori*; Cmed, *Cnaphalocrocis medinalis*; Csup, *Chilo suppressalis*; Gmol, *Grapholita molesta*; Pxyl, *Plutella xylostella*; Slit, *Spodoptera litura*; Msex, *Manduca sexta*; Snon, *Sesamia nonagrioides*. Highlighted clades: pheromone binding proteins, PBPs (purple), general odorant binding proteins, GOBPs (blue), Plus-C OBPs (wathet blue) and Minus-C OBPs (green). Node support (circles at the branch nodes) was estimated using an approximate likelihood ratio test based on the scale indicated at the top left. Bars indicate branch lengths in proportion to amino acid substitutions per site.

Twenty-two unigenes were identified encoding OBPs, including two general odorant binding proteins (GOBPs) and three pheromone binding proteins (PBPs). All unigenes except one (*GmelPBP3*) are full length, and 18 of the full length unigenes encode proteins with a signal peptide (Supplementary Dataset: Sheet 4). A phylogenetic analysis was performed with OBPs containing PBPs and GOBPs from *B. mori*^29^, *M. sexta*^25^, *C. suppressalis*^24^, *C. medinalis*^22^, *G. molesta*^30^, *Spodoptera litura*^31^, *Helicoverpa armigera*^32^ and other lepidopteran species^33^ (Fig. 4). Except the PBP and GOBP clades, other identified OBPs were clustered with at least one lepidopteran orthologue, and were located in different clades. Information including unigene sequences, lengths, best blastx hits and predicted protein domains of the identified OBPs are listed in Supplementary Dataset: Sheet 4.

### Putative chemosensory proteins

**Figure 5:**
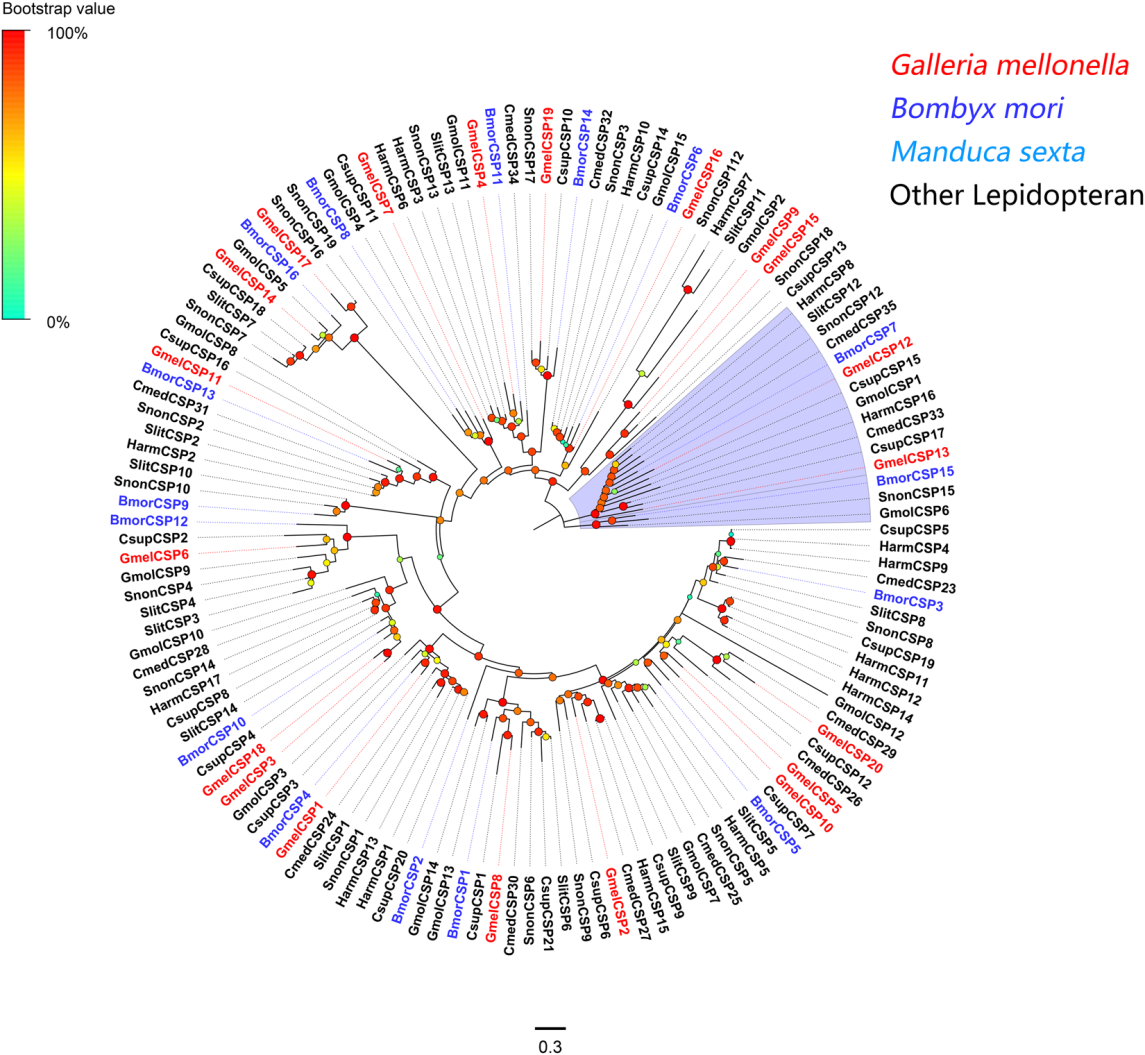
Maximum-likelihood tree of CSPs. The distance tree was rooted by the conservative CSP gene clade (GmelCSP12 and GmelCSP13) (lilac). Species abbreviations: Gmel, *Galleria mellonella*; Bmor, *Bombyx mori*; Cmed, *Cnaphalocrocis medinalis*; Cfum, *Choristoneura fumiferana*; Csup, *Chilo suppressalis*; Gmol, *Grapholita molesta*; Slit, *Spodoptera litura*; Msex, *Manduca sexta*; Snon, *Sesamia nonagrioides*; Harm, *Helicoverpa armigera*; Hvir, *Heliothis virescens*. Node support (circles at the branch nodes) was estimated using an approximate likelihood ratio test based on the scale indicated at the top left. Bars indicate branch lengths in proportion to amino acid substitutions per site.

Twenty unigenes were annotated to encode putative CSPs. Predicted CSP proteins have four highly conserved cysteine residues, which are characteristic of typical insect CSPs (see Fig. S2). Among the 20 CSP-encoding unigenes, 17 were full length and each of the full length unigenes encodes a protein with a signal peptide (Supplementary Dataset: Sheet 5). A phylogenetic analysis was performed with CSPs from *B. mori*^34^, *C. medinalis*, *C. suppressalis*^24^, *Choristoneura fumiferana*, *C. suppressalis*, *G. molesta*^30^, *S. litura*, *S. nonagrioides*^26^. *M. sexta*, *H. armigera*^32,35^ and *H. virescens*. All identified candidate CSP proteins were clustered with at least one lepidopteran orthologue (Fig. 5). Information including unigene sequences, lengths, best blastx hits and predicted protein domains of the 20 CSPs are listed in Supplementary Dataset: Sheet 5.

### Tissue- and sex-specific expression of identified genes

**Figure 6:**
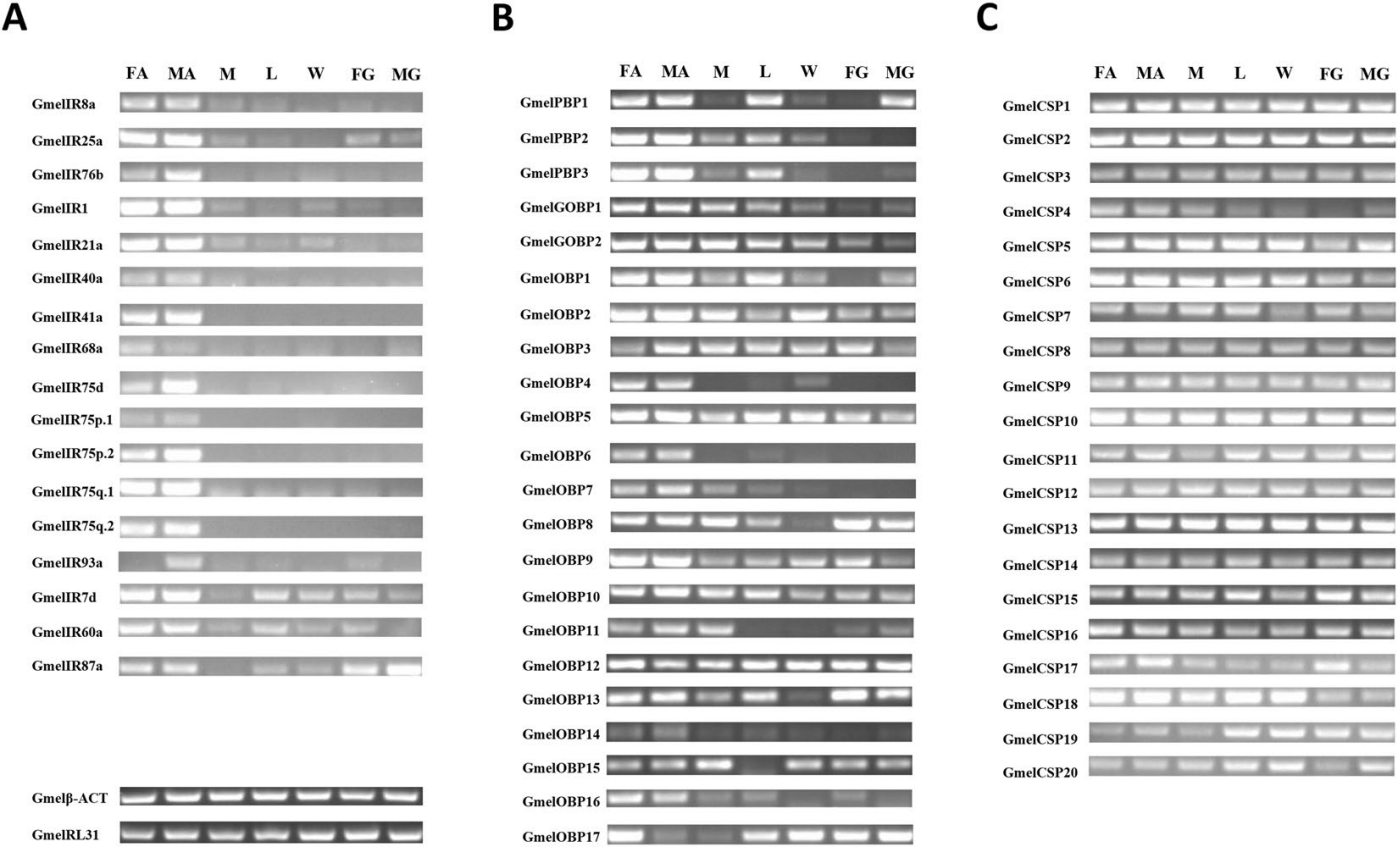
Tissue- and sex- specific expression of iGluRs/IRs, OBPs and CSP. Two reference genes, β-actin (Gmelβ-ACT) and ribosomal protein L31 (GmelRL31) were used as internal references to test the integrity of each cDNA template. Abbreviations: FA: female antennae, MA: male antennae, L: forelegs, W: wings, FG: female ovipositor-pheromone glands, MG, male external genitalia.

To further analyze expression profiles of putative *G. mellonella* OR-, IR-, OBP-, and CSP-encoding unigenes, semi-quantitative RT-PCR was performed using samples derived from seven different tissues, including female antennae, male antennae, mouthparts, forelegs, wings, female ovipositor-pheromone glands and male external genitalias (Figs 6 and S3–S19). All 14 IR-encoding unigenes were predominantly or exclusively expressed in both male and female antennae with three divergent IR-unigenes, *GmelIR7d*, *GmelIR*6*0a* and *GmelIR8*7*a*, expressed at significant levels in other tissues as well (Fig. 6A). Among the unigenes encoding GmelOBPs, expression patterns were different for each gene among different tissues. Only two OBP-encoding unigenes, named *GmelOBP4* and *GmelOBP6*, were almost exclusively expressed in antennae. Three OBP-encoding unigenes, *GmelPBP1*, *GmelPBP2* and *GmelPBP3*, were abundantly expressed in antennae, mouthparts or tarsi. The remaining OBP-unigenes were expressed abundantly in antennae as well as in other tissues (Fig. 6B). For unigenes encoding CSPs, all identified unigenes were expressed abundantly in all analyzed tissues (Fig. 6C).

**Figure 7:**
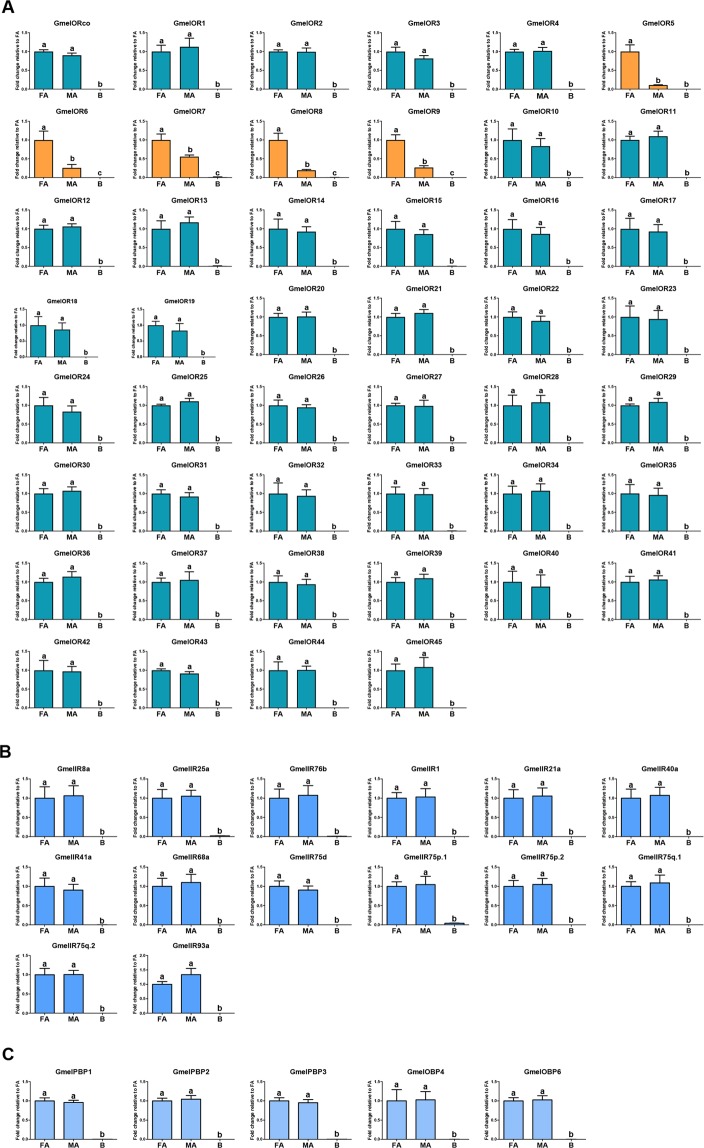
Relative expression levels of all ORs (**A**), antennal IRs (**B**) and the antenna-enriched OBPs (**C**) in the female and male antennae, and other body parts. Abbreviations: FA, female antennae, MA, female antennae, B, other body parts (the pooled tissue mixture of mouthparts, forelegs, wings, female ovipositor-pheromone glands, and male external genitalia). Highlighted histograms: the female-biased ORs (GmelOR6-9) (orange). The expression levels were estimated using the 2^−ΔΔCT^ method. The relative expression level is indicated as mean ± SE (n = 3). Standard error is represented by the error bar, and different letters indicate statistically significant difference between tissues (p < 0.05, ANOVA, HSD).

The expression profiles of the antenna-predominant olfactory-related genes were further analyzed using quantitative real-time PCR (qPCR). Expression levels of all the 62 antenna-dominant unigenes were indeed antenna-predominant (Fig. 7A). Five OR-encoding unigenes, namely *GmelOR5*, *6*, *7*, 8 and 9, were expressed at higher levels in female antennae than in male antennae. The remaining OR-encoding unigenes were equally expressed in the antennae of both sexes. A similar expression pattern was also found for the gene *GmelOrco*. For the IR-encoding unigenes, all 14 unigenes were predominately and equally expressed in male and female antennae (Fig. 7B). Predominant and equal expression levels were also found for the OBP-encoding unigenes (Fig. 7C).

## Discussion

During the last few decades, chemosensory gene families have been extensively studied in lepidopteran insects, resulting in substantial understanding of the molecular mechanisms for pheromone perception in these insects. A common phenomenon for these model lepidopterans is that females produce and release sex pheromones to attract males for mating. However, the moth *G*. *mellonella* displays a unique mating behavior. During the mating period, it is the males that produce and release sex-pheromones to attract females. This is contrary to the general observation that females produce sex pheromones that can attract conspecific males in most lepidopterans. The uniqueness associated with this lepidopteran species may provide opportunities to gain insight into the molecular mechanisms that are associated with olfactory behavior. From this point, our studies here provide a foundation for future studies that may lead to a better understanding of the molecular mechanisms associated with male-dominant pheromone perception in insects. Specifically, the chemosensory-related unigenes identified and characterized here are useful targets for further studies to reveal chemical perception in this economically important and biologically unique species.

Because of the apparent differences in sexual behavior between *G. mellonella* and other well-known moths such as *B. mori*^15^, *C. pomonella*^36^ and *M. sexta*^37^, phylogenetic analyses were conducted with genes from these species. No orthologs for the unigenes in the Lepidoptera PR clade were identified in *G. mellonella*. The absence of PRs in the antenna of *G. mellonella* may be related to the type of pheromone compounds used. Additionally, five OR-encoding unigenes, namely unigenes encoding GmelOR5, 6, 7, 8 and 9, showed female-biased expression. The female-specific OR unigenes might be involved in detecting sex pheromones released by males. Alternatively, the female-biased OR genes might be responsible for other female specific behaviors such as finding bee-comb hosts for oviposition. Phylogenetic analysis showed GmelOR5, 6, 7, 8 and 9 distributed in different clades, and might respond to the multi-component pheromone in *G. mellonella*^14^ (Fig. 1). Since our analysis was not comprehensive, it is likely that not all OR-encoding unigenes were identified in this research even though the sequencing quality in our project was comparable other reports. In addition, two lepidopteran-specific GOBP orthologs and three lepidopteran-specific PBP orthologs were also identified in *G. mellonella* in this study. It has been suggested that PBPs and GOBPs may be mostly likely associated with pheromone-sensing and plant volatile-sensitive detection in most lepidopteran insects, respectively^2^. Moreover, the PBPs that involved known male pheromones in most lepidopteran species also showed antenna-predominant and male-biased expression^26,32,38,39^. However, three PBPs (*GmelPBP1*, 2 and 3) in *G. mellonella* were also expressed in gustatory organs including mouthparts or tarsi in addition to high levels of expression in antennae (Fig. 6). Thus, these PBPs in *G. mellonella* could potentially participate in other physiological processes, or these PBPs may not only perform functions in antennae, but may also be involved in sensory functions in other organs. Additionally, the qPCR indicated *GmelPBP1, 2* and 3 equally expressed in both males and females (Fig. 7). We speculated that they are not involved in male pheromone perception. Obviously, molecular characterization and expression profiles in both ORs and OBPs in the *G. mellonella* is distinct from other lepidopteran insects, which may imply that those attributed to male pheromone perception.

The second type of olfactory receptor, IR, is a conserved family that functions as receptors for the detection of amine acids, general odors, sex pheromones, gustation, thermosensation and hygrosensation^8,40–52^. According to functional studies of antennal IRs in *D. melanogaster*, IR40a is required for response to the insect repellent DEET^8^, and IR64a is acid sensitive^40^. IR76b is co-expressed with IR41a to mediate long-range attraction to odor^48^. IR21a and IR25a mediate cool sensing^51^. IR93a and IR68a are involved in physiological and behavioral responses to both temperature and moisture cues^50,52^. In the ML tree, putative *G. mellonella* IRs were clustered with *D. melanogaster* IRs and other known lepidopteran IRs. The IRs orthologs in *G. mellonella* might have similar sensory functions. In addition to these Drosophila IR paralogs, we also identified several clades that are lepidopteran-specific antennal IRs, including IR1, IR75p and IR75q. The function(s) of these lepidopteran-specific antennal IRs remain unknown. Based on our results, *GmelIR1*, *GmelIR75p* and *GmelIR75q* were predominantly and equally expressed in antennae of both females and males. Further investigations are needed to reveal the functions of the lepidopteran-specific antennal IRs.

In the antennae of *G. mellonella*, we identified 20 CSPs and all were found abundantly expressed in all tissues examined. CSPs function as carriers for odorant molecules^3^. Our results suggest that *G. mellonella* CSPs could also be involved in non-sensory functions. Genes in the SNMP1 subfamily are usually expressed in pheromone-sensitive olfactory sensory neurons, and mediate responses to the lipid pheromones^12,13,53,54^. Genes in the SNMP2 subfamily in moth are specifically expressed in supporting cells with functions unknown^55–58^. Two putative SNMPs were identified from the *G. mellonella* transcriptomes, with one belonging to SNMP1 and the other with similarity to SNMP2. These two *G. mellonella* SNMPs may play the same role as in *D. melanogaster* and other moths.

## Conclusion

We conducted a transcriptomic analysis on the greater wax moth, *G. mellonella* using high-throughput sequencing and annotated gene families potentially involved in chemoreception. Based on the transcriptomic analysis, a repertoire of 46 ORs, 25 IRs, 2 SNMPs, 22 OBPs and 20 CSPs were annotated. Then, tissue- and sex-biased expression levels of the differentially expressed genes were analyzed by RT-qPCR and qPCR. The expression profile analysis revealed that all 46 ORs, nine IRs, two OBPs were primarily expressed in antennae and some of these genes were sex-specific. Identification of the chemosensory genes and initial analyses on their expression profiles should facilitate functional studies in the future on chemoreception mechanisms in this species and related lepidopteran moths.

## Methods

### Insect rearing and tissues collection

*G. mellonella* was reared on artificial diet^59^. Adult moths and larvae were maintained in the dark at 30 °C and 60% relative humidity. Once they emerged as adults, 2-day-old virgin adults were used for tissue sample collection. Different tissues were dissected and stored at −80 °C until use. For transcriptome sequencing, female antennae (n = 100) and male antennae (n = 100) were individually collected. For Semi-quantitative RT-PCR analysis, female antennae (n = 100), male antennae (n = 100), mouthparts (n = 100), forelegs (n = 80), wings (n = 80), female ovipositor-pheromone glands (n = 60), male external genitalia (n = 60) and other body parts (the pooled tissue mixture of mouthparts, forelegs, wings, female ovipositor-pheromone glands, and male external genitalia, n = 10 each) were collected separately and two replicates were generated for each tissue set. For qPCR analysis, emale antennae (n = 100), male antennae (n = 100) and other body parts (the above pooled tissue mixture) were collected separately and three replicates were generated for each tissue set.

### RNA extraction

Total RNAs were separately extracted and purified using TRIzol reagent (Invitrogen, Carlsbad, CA, USA). The integrity of total RNA was assessed with the Bioanalyzer 2100 system (Agilent Technologies, USA), and the purity was detected on a NanoDrop 2000 spectrophotometer (Thermo Scientific, USA). RNA concentration was accurately measured using Qubit flurometer (Life Technologies, USA).

### Sequencing and *de novo* assembly

Transcriptome sequencing was performed by Novogene Bioinformatics Technology Co. Ltd (Beijing, China). Three micrograms of total RNA from antennae of females and males, separately, were used for the synthesis of duplex-specific nuclease-normalized cDNA. cDNA libraries were prepared using Illumina’s sample preparation instructions (Illumina, San Diego, CA). Libraries were then sequenced using the Illumina HiSeq4000 system (Illumina Inc. San Diego, CA) to obtain 150 bp paired-end reads. The raw sequence transcriptome data have been submitted to the NCBI Short Read Archive (SRA) database under accession numbers SRX5010722.

The raw reads were filtered to remove reads containing unknown (poly-N) or low-quality and adaptor sequences using the FASTX toolkit. Trinity *de novo* program (version r2013-02-25) with default parameters was used to assemble the clean read sequences from males and females into transcripts^60,61^. Then the Trinity outputs were clustered using TGICL^62^. The consensus cluster sequence and singletons compose the final unigenes dataset. The longest copy of redundant transcripts was selected as a unigene.

### Identification of chemosensory genes

tBlastn searches (Geneious software) were performed with the available sequences of OR, IR, SNMP, OBP and CSP from other insect species (searching NCBI databases with keywords “odorant receptor AND insecta”, “ionotropic receptor OR ionotropic glutamate receptor AND insecta”, “sensory neuron membrane protein AND insecta”, “odorant-binding protein AND insecta” and “chemosensory proteins AND insecta”) as “query” to identify candidate chemosensory genes unigenes in *G. mellonella* antennal transcriptomes. Open reading frames (ORFs) of candidate chemosensory genes unigenes were predicted in the ORF finder tool at the National Center for Biotechnology Information (NCBI), and translated to amino acid sequence in Geneious (version 9.1.3.). In addition, Protein domains (e.g. transmembrane domains, signal peptides, secondary structures, etc.) were further predicted by queries against InterPro using the InterProScan tool plug-in in Geneious (version 9.1.3.)^63^. Candidate chemosensory unigene names were represented by a four-letter species abbreviation combined with the name of other Lepidoptera insect orthologs (based on phylogenetic analysis)^27,64^, and given the same name (e.g. SnonORco, GmelORco, SnonIR8a, GmelIR8a, BmorSNMP1, GmelSNMP1, SnonGOBP1, GmelGOBP1).

### Phylogenetic analysis

Amino acid sequences used for phylogenetic tree construction were aligned with the MAFFT alignment tool (E-INS-I parameter) plug-in in Geneious (version 9.1.3.)^65^. Phylogenetic reconstruction with the maximum-likelihood (ML) method was performed in FastTree (version 2.1.7) using the Jones, Taylor, Thornton substitution model^66^ with 1000 bootstrap replications. Phylogenetic trees were visualized with FigTree (http://tree.bio.ed.ac.uk/software/figtree).

### Tissue- and sex- specific expression analysis

iGluRs/IRs, OBPs and CSPs are extensively expressed in diverse tissues in insect^3,27^, therefore semi-quantitative RT-PCR was employed to compare the expression of these genes in different tissues including female antennae, male antennae, mouthparts, forelegs, wings, female ovipositor-pheromone glands and male external genitalia, to define the antenna-predominant candidates. Total RNA from the analyzed tissues was extracted as described above and treated with DNase I (Takara, China) to remove trace amounts of genomic DNA. cDNA was synthesized from total RNA using PrimeScript RT reagent Kit (Takara, China). The PCR reaction programs were as follows: 95 °C for 2 min, followed by 35 cycles of 95 °C for 30 sec, 56 °C for 30 sec, 72 °C for 1 min, and a final extension for 10 min at 72 °C. PCR amplification products were separated on a 1.5% agarose gel. Two reference genes, β-actin (Gmelβ-ACT) and ribosomal protein L31 (GmelRL31) from *G. mellonella* antennal transcriptomes were used as the control genes to assess the cDNA integrity (see Supplementary Dataset: Sheet 6). Two repeats with two biological samples of each gene were performed in this experiment, to ensure the results consistent for the two biological replicates. All specific primers used in our study were designed in Primer3web (version 4.0.0) (http://primer3.ut.ee/) and listed in Table S2.

ORs are mainly expressed in insect antennae^67,68^, therefore the expression profiles of all ORs, antennal IRs and the antenna-enriched OBPs were analyzed using quantitative real-time PCR (qPCR). Total RNA and cDNA synthesis were performed in samples as described above, including female antennae, male antennae and other body parts. The qPCR was performed on a LightCycler 480 system (Roche Applied Science) with a SYBR Premix ExTaq kit (Takara, China). The reaction programs were as follows: 95 °C for 15 min, followed by 40 cycles of 95 °C for 10 sec and 60 °C for 32 sec. Then, the PCR products were heated to 95 °C for 15 sec, cooled to 60 °C for 1 min, heated to 95 °C for 30 sec and cooled to 60 °C for 15 sec to measure the dissociation curves. All specific primers for qPCR are consistent with semi-quantitative RT-PCR experiment. Two reference genes, Gmelβ-ACT and GmelRL31 (Table S2) were used for normalizing the target gene expression and for correcting for sample-to-sample variation. Negative controls without template were included in each experiment. For each gene, three biological replications were performed with each biological replication measured in three technique replications. Relative quantification was performed with the 2^−ΔΔCT^ method^69^. All data were normalized to reference genes levels from the same tissue samples and the relative fold change in different tissues was calculated with the transcript level of the female antennae as calibrator. The comparative analyses of each target gene among different tissues were determined using a one-way nested analysis of variance (ANOVA) followed by Tukey’s honest significance difference (HSD) test using Prism 6.0 (GraphPad Software, CA). Values are presented as mean ± SE.

## Supplementary information

Supplementary Information

Supplementary Dataset
