# Supplementary material for: Candidate chemosensory genes identified from the greater wax moth, Galleria mellonella, through a transcriptomic analysis
Source: Sci Rep. 2019 Jul 11;9:10032. doi: 10.1038/s41598-019-46532-x (PMC6624281; doi:10.1038/s41598-019-46532-x)
Supplement: Supplementary file 1 — Supplementary Information [file 41598_2019_46532_MOESM1_ESM.pdf]

Supplementary Information for

Candidate chemosensory genes identified from the greater wax moth, *Galleria mellonella*, through a transcriptomic analysis

Hong-Xia Zhao[1], Wan-Yu Xiao[2], Cong-Hui Ji[1], Qin Ren[3], Xiao-Shan Xia[1], Xue-Feng Zhang[1*], Wen-Zhong Huang[1*]

[1]Guangdong Key Laboratory of Animal Conservation and Resource Utilization, Guangdong Public Laboratory of Wild Animal Conservation and Utilization, Guangdong Institute of Applied Biological Resources, Guangzhou 510260, PR China

[2]Guangzhou Academy of Agricultural Sciences, Guangzhou 510308, PR China

[3]Chongqing Academy of Animal Science, Chongqing 402460, PR China

[*]Corresponding author

Email addresses:

Hong-Xia Zhao: hxzh110@126.com

Wan-Yu Xiao: yuwanxiao22@126.com

Cong-Hui Ji: 94660538@qq.com

Qin Ren: 53665069@qq.com

Xiao-Shan Xia: 244090974@qq.com

Xue-Feng Zhang: zxf3802@126.com

Wen-Zhong Huang: gggbmm@163.com

Table S1. Overview of the sequencing and assembly process.

	MA (male)	FA (female)
Mean length (bp)	150	150
Total Raw Reads	37,238,352	41,665,706
Total Raw Bases	5,585,752,800	6,249,855,900
Total Clean Reads	36,840,810	41,460,680
Total Clean Reads Ratio (%)	98.93	99.51
Total Clean Bases	5,526,121,500	6,219,102,000
Total Clean Bases Ratio (%)	98.93	99.51
Total Adapter Reads	381,922	203,664
Total Adapter Reads Ratio (%)	1.03	0.49
Total Low Quality Reads	1,562	1,362
Total Low Quality Reads Ratio (%)	0.04	0
Clean Reads GC (%)	42.2	43.42
Clean Reads Q20 (%)	93.15	96.1
Clean Reads Q30 (%)	84.65	90.78
Combined Trinity assembly of the male and female antennal transcriptomes		
	Unigenes	Transcripts
Total Number	54,234	65,593
Min Length (bp)	200	200
Max length (bp)	29,406	29,406
Mean Length (bp)	1,125	1,388
Unigene N50	2,131	2,511
Unigene N90	418	492

Table S2. Primers used in qPCR.

Gene Name	Forward Primer (5'-3')	Reverse Primer (5'-3')	TM (℃)	Product size (bp)
Reference genes				
Gmelβ-ACT	GGACTTGTACGCCAACACAG	CCACATCTGCTGGAATGTCG	59	196
GmelRL31	TGCACAAACGTCTTCATGGT	GGGTGAACAGTTTATGGGCA	59	228
ORs				
GmelOrco	GCTCACTGCGAACACAATCA	CAGCAGCCTCCTCATTTTCG	59	190
GmelOR1	CGGCTTGCAGTTCCCAGTAT	AAAGAGCCATCCAGTCCACA	59	210
GmelOR2	CAGAACTTCTTCGAGCCCTG	ACTTCGATTTTCCCACGGTG	59	231
GmelOR3	GTGGGCATCATTGTCGGTTT	CGATCCACCGGCATACAAAG	59	237
GmelOR4	CGCAACTTCTCTCCAGCATG	GGCGACATCTGTTCCCATTT	59	220
GmelOR5	GCAGCTAAAGTCCTGATGATGT	CCAAAGTTGCCCAAAATCGC	59	173
GmelOR6	ACCGTCATTAGCAAAGCGTT	CAAAGGAACGGCAGTACACC	59	158
GmelOR7	AGACAACGTGGGAAGGCTAT	CAATCTGCCTGTGGTGTACC	59	210
GmelOR8	GTCGCTGATAGTTTGGAGCA	AAGTACCATGTCAGTGCGGA	59	198
GmelOR9	TGTGTGCAGATGTGGAGAGT	CCATCCGCAGTTGTACAGTG	59	236
GmelOR10	GACGTTATCAGCGGACCCTA	GGTACATAGAAGAGCACCGC	59	194
GmelOR11	TGAGGACGGTGAAGAAACGA	AGGTGCAGGACGTATGTTGA	59	208
GmelOR12	GCAGTTTCCTTGTTGTTCGC	CTGGTTTCTGAGCCTGCATC	59	178
GmelOR13	CAGACTGACGACATCTCCGA	TCAATCATAGGTTGTACTCCCGT	59	158
GmelOR14	TGCCTTCTCTGTGGTGAACT	GACACGCTAACACTCTCCCT	59	192
GmelOR15	TGGTAGTAGAGGCTTGGCTG	GCTCATCATCATTCCCTGGC	59	219
GmelOR16	TGACCAGTGCTGCTTTTCTG	GCCCCATCCTATTACCGTCA	59	230
GmelOR17	GGCTTCGTGTTCCAGTTTGT	CGTCTCCTTTTGCATCGTGT	59	160
GmelOR18	GCGTCACAGCCTATCAGATT	AACACTCTCATACGGACCCC	59	164
GmelOR19	TACGCCAGCCAGTTTTAACG	GACACCATCGCAATGCCTAG	59	180
GmelOR20	GTTGCTGATGAAGAAGGGCC	GAACCACATGTCGAATGCCA	59	210
GmelOR21	TTGCCTGATTCAGCTGCTTC	AACTTGCACGTAGAATCGCG	59	227
GmelOR22	TACTTTGCCCTCGCCCTTAT	TCGTCAAGTCCCGTGATCAT	59	191
GmelOR23	TTTGCCAGACACGGGTTTAC	TGATTTTGCCAGAACACGCA	59	195
GmelOR24	GCAGCGGCCAATATAAACGT	GCACAGGCGAACATGATTGA	59	221
GmelOR25	GGTTTGCTTGTGTGTGTTGC	TGATCAAGTTCACCGCAGAG	59	246

GmelOR26	CGCACCCCATTCTCGTAGTA	ACAGATAACGCCACGCTAGT	59	184
GmelOR27	CATTGTTGCCAGTACTCCCG	ATTCCGGGCAGAAAACACAC	59	199
GmelOR28	CGTTGATCCTAGCACCGTTG	TCGCTACAATACCACCTCCA	59	192
GmelOR29	CACACTTGCCACCTTGCTTA	TCAGTGCTCACCATAACCCA	59	249
GmelOR30	CGTAGCTTATGACTGCCTGC	CTATCCCACACCTTCACCCG	59	250
GmelOR31	GGTTACTTAGCCACAGTCGC	GCGTTCATCATTTTCACCGG	59	192
GmelOR32	TGCAGGATATAATAGCGAGTGGA	CCATCTTGCCGGTCTTGAAC	59	201
GmelOR33	TTACTAAGGAGAGGTGGCCG	GCTCGATAAGTTCCTGATGCC	59	228
GmelOR34	GTTACCTACTACCCGACGCA	AACTTCTCCGTTGCTTGCTG	59	242
GmelOR35	CGCGTTTCAACTAGTCTCGG	CAAGGACTGAAGTATGCCGC	59	162
GmelOR36	TCTGACCAGGACGCTGAAAT	TGTGCTGTGTTTGTTGACGA	59	179
GmelOR37	ATGAGATCGTTGGTCCGCAA	ACCAGGAACCAAGTCGAAAA	59	208
GmelOR38	AAACATCCGGCCTAAAACATTT	ACAGACAGGCTTTAAATATGCGT	59	159
GmelOR39	CGATGGCTATGTACCTGGGT	GAGGATTTCAGCACCGCAAT	59	247
GmelOR40	ACACTGCTTGCACCATGTTC	TAAGAACCTGCGCGAAACAC	59	159
GmelOR41	GTGGTTCAATTCACGTCGAGT	TCACTTTTCCACCAATCACTTGT	59	173
GmelOR42	ACACCAAAGAAGATCGAAATGCT	TCGCGTTAATTTTCCAGCCC	59	220
GmelOR43	CGATTCCGTTGTTTGCTGAGA	GCTCGCCTTAAGAACAGAAGT	59	233
GmelOR44	ATTTCACCCACCCCTACCTG	CCTGTCGTAATCACTTGCCA	59	235
GmelOR45	GCGGCTCAACAGTTCATCAA	TGGGCCACATTTCTCTCAGT	59	187
Antennal IRs				
GmelIR8a	TATTGGAATGACACAGCGCG	TCTTGTTTTCCGCCGGTTTT	59	214
GmelIR25a	AGAGACGATGATGCCCCAAA	ATTTTCCCTCTCCGCCATCA	59	222
GmelIR76b	ATCAGGAGGCATGGTAAGCA	TAGAGGAATGCTGACGGTGT	59	242
GmelIR1	AACGGGGATTTTCTGCGTTC	CCTGCAACCAAGATCTCGTG	59	164
GmelIR21a	GATCGTGGTGGTTGGGAAAC	GCCAAACGGTGCAAAACATT	59	249
GmelIR40a	GGCCATCATCAGACCGTTTC	GAGTCGTCGTGAACCAACAG	59	167
GmelIR41a	CCCCAGGATTCTAGCCAGTT	GCCATGTTGAACCAGCAGAG	59	194
GmelIR68a	ACTGTACCCTTTCCCAACCC	ATCGTCACCACTACAGAGGG	59	176
GmelIR75d	CCTCAGCATCGTCGAAGGTA	TTTCTGATTGGCACGCTGAC	59	171
GmelIR75p.1	CAATGACTGACCGCTTCGAG	TTCCTGACCTCCCATTTGCT	59	205
GmelIR75p.2	GAAGGATGCCAGGTCGAGTA	GCAGGACTTAGCACGAATCG	59	196
GmelIR75q.1	TGGACATCGTTATCTGGGCT	TAGCTTTTGTCGCAGTGTCG	59	175
GmelIR75q.2	TCTGCAAACATTGTCGCACT	TGACACCTTCTTCCATGCTCA	59	217

GmelIR93a	GCAGTCTCCGGCTATAGAGG	TTTCGCCACCACTGACAATG	59	174
Divergent IRs				
GmelIR7d	CGGTTGGGGATCACTACGTA	AAGAACGGCCACTTGAGTTG	59	241
GmelIR60a	GTGTTGACAGTGACCGCTTA	ACATCGATCAATGCTTGGGT	59	224
GmelIR87a	GTGTGTTCCTAAAGCTGGCC	TACCGCTTCTTGGCTCGAAT	59	230
OBPs				
GmelPBP1	CCTCTCCGCAAAACTGAACC	AGAATCATGTCAGGTGCCCA	59	237
GmelPBP2	TACAGAAGATCGGATGCACC	AGAAGTTCCTCCATCGACGG	59	216
GmelPBP3	GCAATTAGCGTGAGGGATGT	CACTCGTGTCCACCAAGTTT	59	224
GmelGOBP1	GTGCTTGTGATCAGTCTGCC	AGTCGGTGAGGAGGTTGAAG	59	226
GmelGOBP2	AGTTCATCGGGAGTTGGGTT	GCAGCGGACTTCATTACACG	59	219
GmelOBP1	GTGTTGGCTTTGGAGGGAAA	GGTTCAATTCCTCTACCACGC	59	192
GmelOBP2	ATGCCCGACCCTAAACTCAA	TGCCAACATACTTGCGTGAG	59	206
GmelOBP3	AAAGCAGCCAAAGAATCCGG	ACCGTCTGTCACTTCTTCGT	59	219
GmelOBP4	CGGAGTAGCGAAACTGAACTG	TCCTGCGAAAATTACTGCCG	59	219
GmelOBP5	TGGGATGACACGACAACAAC	CATCCTTCATCTCTGCGGGA	59	245
GmelOBP6	GGTTCACGCTTGGAATTTTCG	GCACATAACGTCTCTGTCTTCG	59	167
GmelOBP7	ATGCTTTGAACTGTCGCTCC	TTCCCATTCTGTCACGGTCA	59	210
GmelOBP8	AAGCGATCAAAGAATCCGGC	GATCTAATGCACGCTGGGTC	59	238
GmelOBP9	GGAAACAGTGCCAACCCAAA	GACTTTCCTGCAAGCAACGA	59	236
GmelOBP10	GATCACGAGAAGTTAGCGGC	TCCGTCTGATTCGTCCCAAA	59	201
GmelOBP11	AAATACGAACAACCAGCGCC	TTCACCTCCACTCCCTGTTC	59	181
GmelOBP12	TGTAATGGTTATCGGGCTTGC	ACGTCCATTCCTTTTGCCATA	59	232
GmelOBP13	CCATCGACGAGTTGAAGCAG	TTCCAGCCTCGTCCATCATT	59	177
GmelOBP14	TGGTATTGTAGATGGCGAAGGA	CGTACCCTGCGAATAACACT	59	172
GmelOBP15	GGGGATACTTCATGGCCGTA	CCAGAGTTGCCGCAGTATTC	59	166
GmelOBP16	TTGAGAACCCGGAAGCTACC	TCTTCTGCACCTCTTCCTCG	59	160
GmelOBP17	TACGACCGTCTGTGTGTTCA	CGGTCCTTGGGTCTGAAGAT	59	190
CSP				
GmelCSP1	AAGAGGCTATTGAGACCGGC	GTTCGCCTTTGCCCTATCTT	59	173
GmelCSP2	CCCAAAACGAGCAGTACACC	CGTTGTTGATGAGGTGACCG	59	234
GmelCSP3	TATCTGTCGTGGTAGTGGCG	TCTGGAGTGCATCGACCTTT	59	154
GmelCSP4	CCGGTATGATGATTTCGACGTA	TCCACATGATGTTTCTAAGGCT	59	151
GmelCSP5	CTTTGTGGTCGCAGTTGTGG	TTGCGTTGCTTTTCCGTACA	59	219

GmelCSP6	TGATGGTGTAAATTTGGACGAGA	GTCGGTGATCAATGATGTATCCC	59	209
GmelCSP7	CACAAGACACGTACAACCCG	TGCCGCATGTAGTTTGTAGC	59	171
GmelCSP8	GGAAACTGGACGATGCACTG	TTGGTTAACATCTGCTGCCA	59	168
GmelCSP9	TCATTCTCGCCATTGTCGTG	TTTTGTTCTGGTGTGCAAGAG	59	190
GmelCSP10	GATGAGTTTAGCAGCCGTGG	CCTCCTTGATGTGCAGCTTT	59	182
GmelCSP11	GTGTTTACTTGGAGCCGCAA	TGCTTACCCATCTTCTTTTGCT	59	228
GmelCSP12	CACAAACTGCAATGACGTTAGC	CCCTCTGGATCGAACTTCTTTC	59	177
GmelCSP13	AAGACTGCCACACTCAAACG	GCTGATTCTAAAGCGTCGAAC	59	188
GmelCSP14	GCCCTAGACGATGCTCTCAA	TGTTTGGTTTCTTGCGGTGT	59	161
GmelCSP15	TGTCTTTGGCTACGACGAAA	CTTTGGATCGGGCTGCATTT	59	192
GmelCSP16	TTTGTGTTGTCTGGTGGCAA	ACGGTTTCTACAGCTTCAGGT	59	192
GmelCSP17	GGAGCGATGATGAATTTACCGA	GCGGTGCCAATGTTCTCAAT	59	166
GmelCSP18	GTGCACTCCAGACGGTAAAG	TGCTTTCAGGATCGTATCTACCT	59	173
GmelCSP19	TCCTGCTCTACTTGTGTGCA	CTGGATTACAACGCCCACAG	59	222
GmelCSP20	GCTCACGGCGGCATATATTA	TCCACCCAGTAAGCGTTTTC	59	186

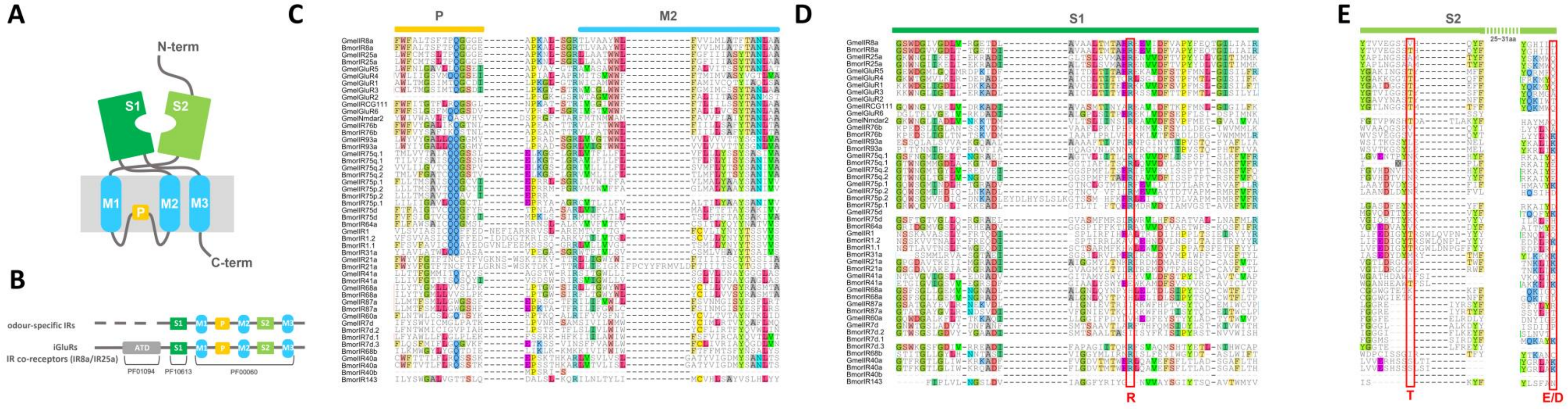

Figure S1. Excerpts from the amino acids alignment showing the predicted iGluRs/IRs binding domains. Predicted protein domain organization of IRs and iGluRs/IR co-receptors illustrated in diagram (A) and linear (B) form with associated Pfam predicted domains. (C) MAFFT amino acid alignment of the ion channel pore (P) and second transmembrane (M2) domains of *G. mellonella* IRs and *Bombyx mori* IRs and iGluRs. MAFFT amino acid alignments of the S1 (A) and part of S2 (B) ligand binding domains of candidate *G. mellonella* IRs and *B. mori* IRs and iGluRs. The key binding residues in iGluRs are in red boxes

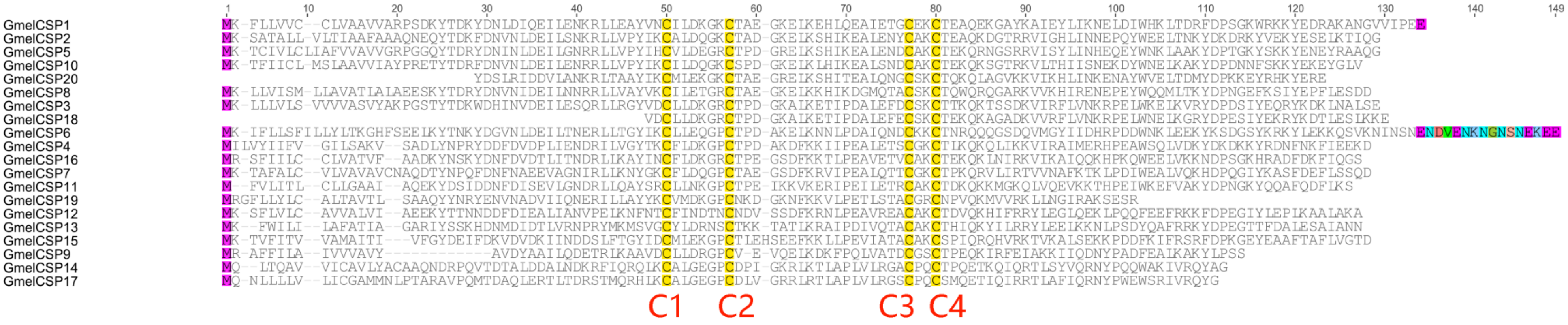

Figure S2. Excerpts from the amino acid alignment showing the predicted CSPs.

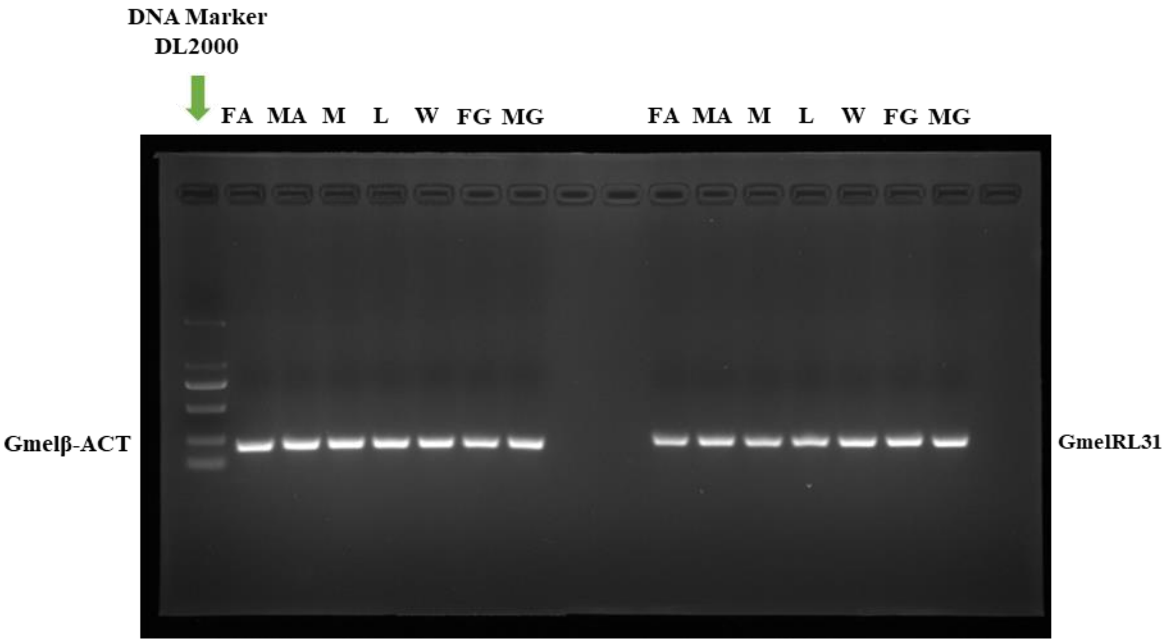

Figure S3. The full-length gel of Gmelβ-ACT and GmelRL31

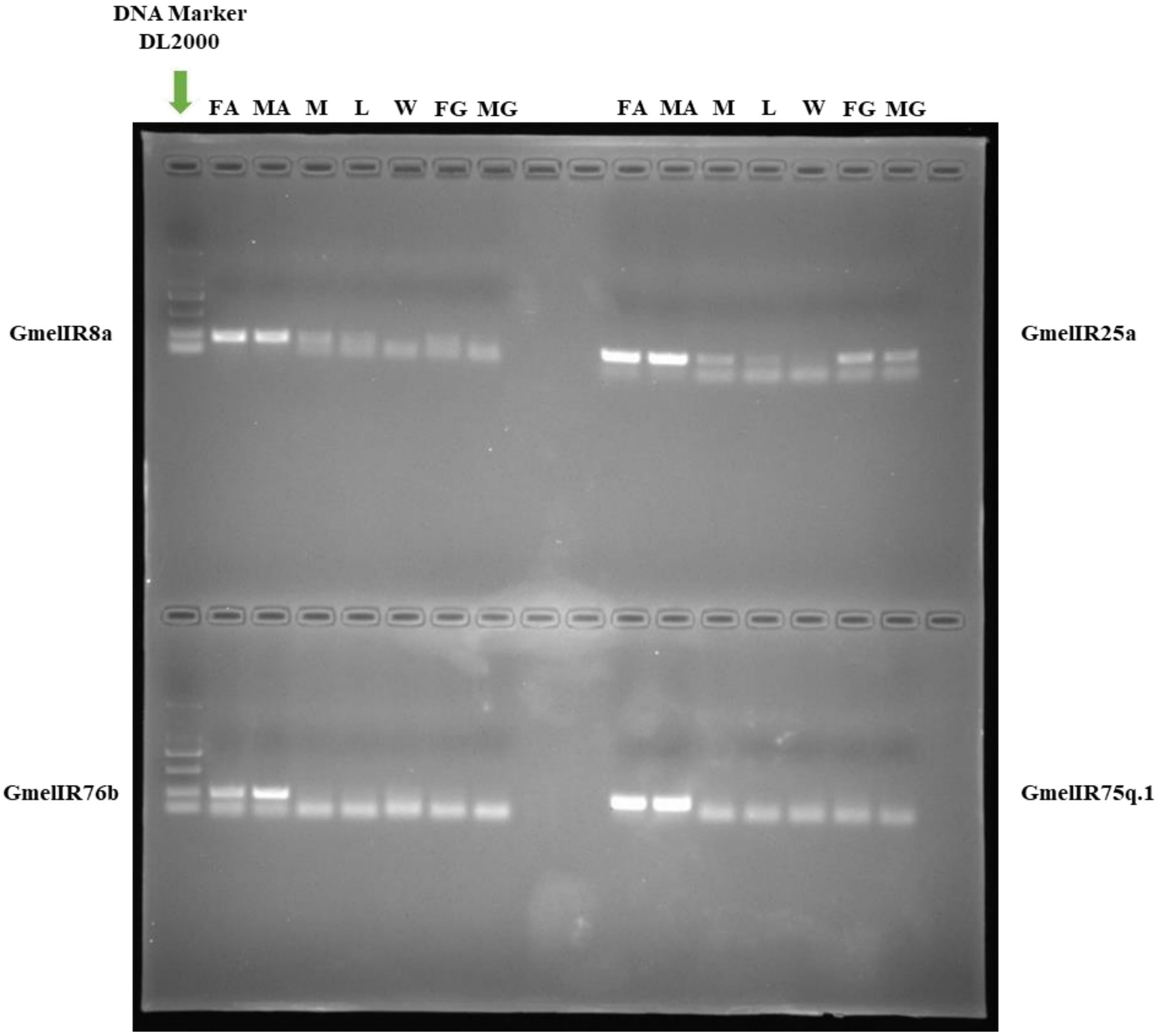

Figure S4. The full-length gel of GmelIR8a, GmelIR25a, GmelIR76b and GmelIR75q.1

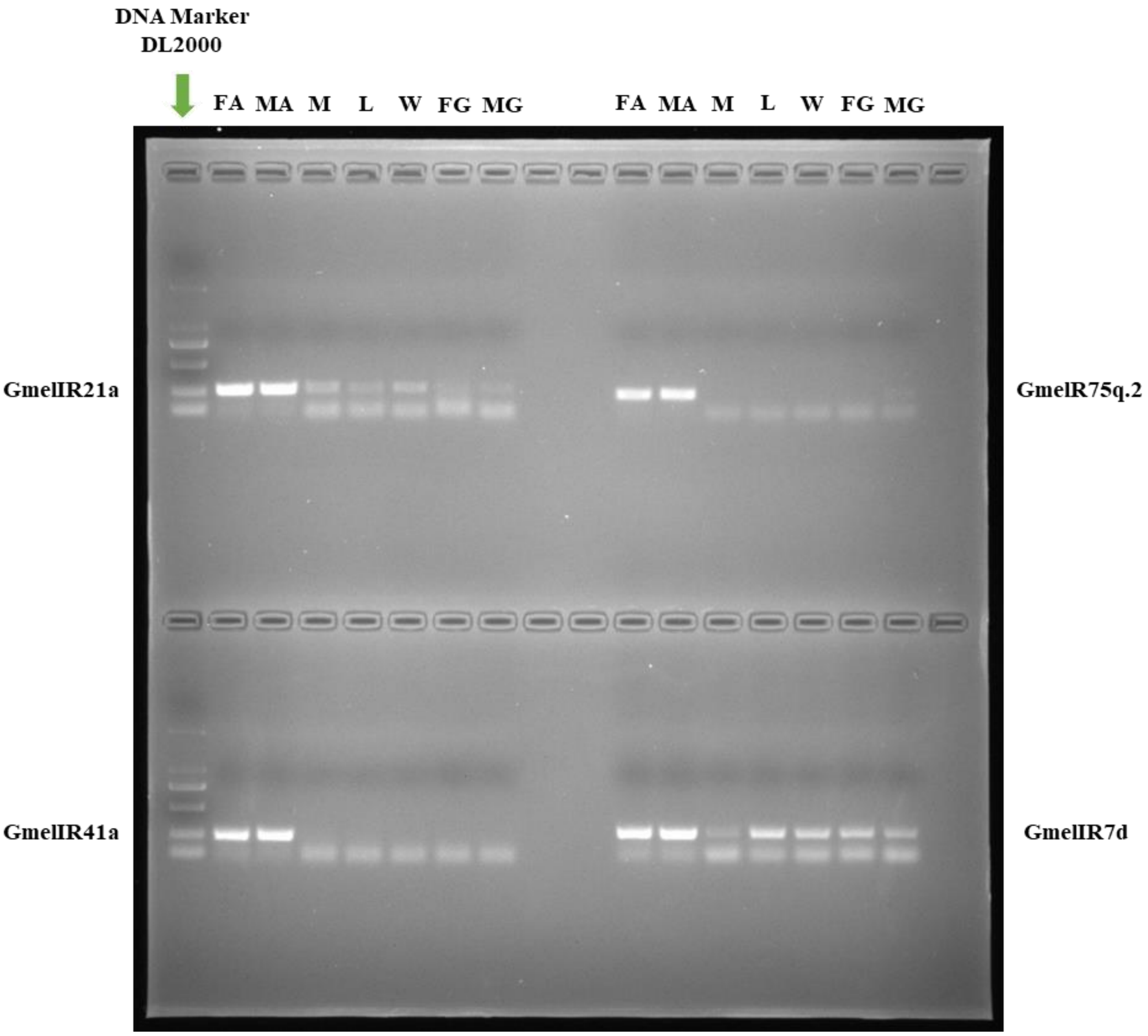

Figure S5. The full-length gel of GmelIR21a, GmelR75q.2, GmelIR41a and GmelIR7d

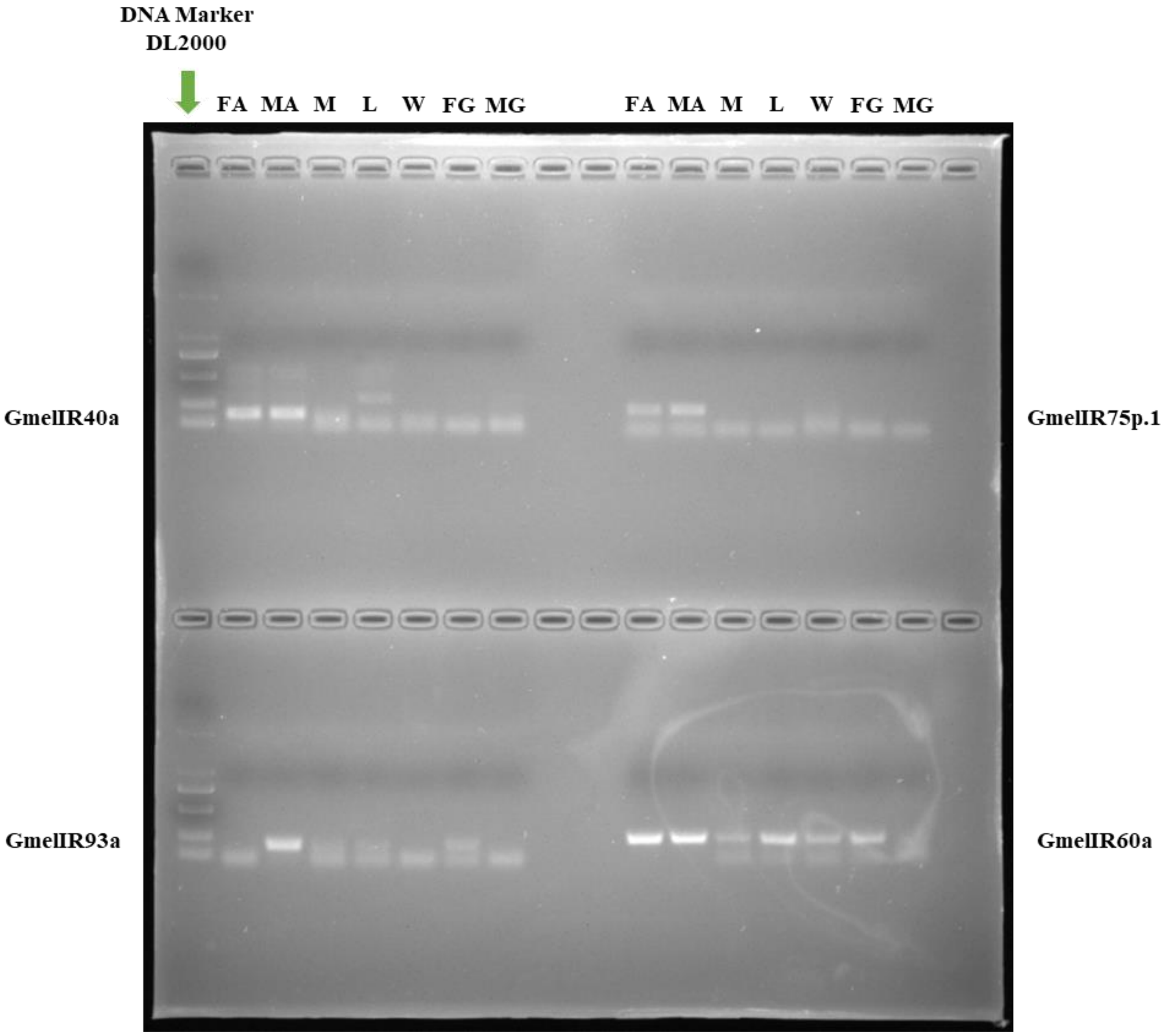

Figure S6. The full-length gel of GmelIR40a, GmelIR75p.1, GmelIR93a and GmelIR60a

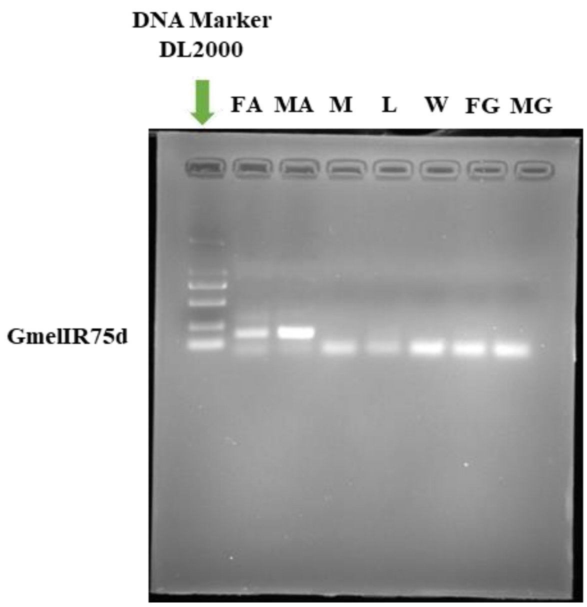

Figure S7. The full-length gel of GmelIR75d

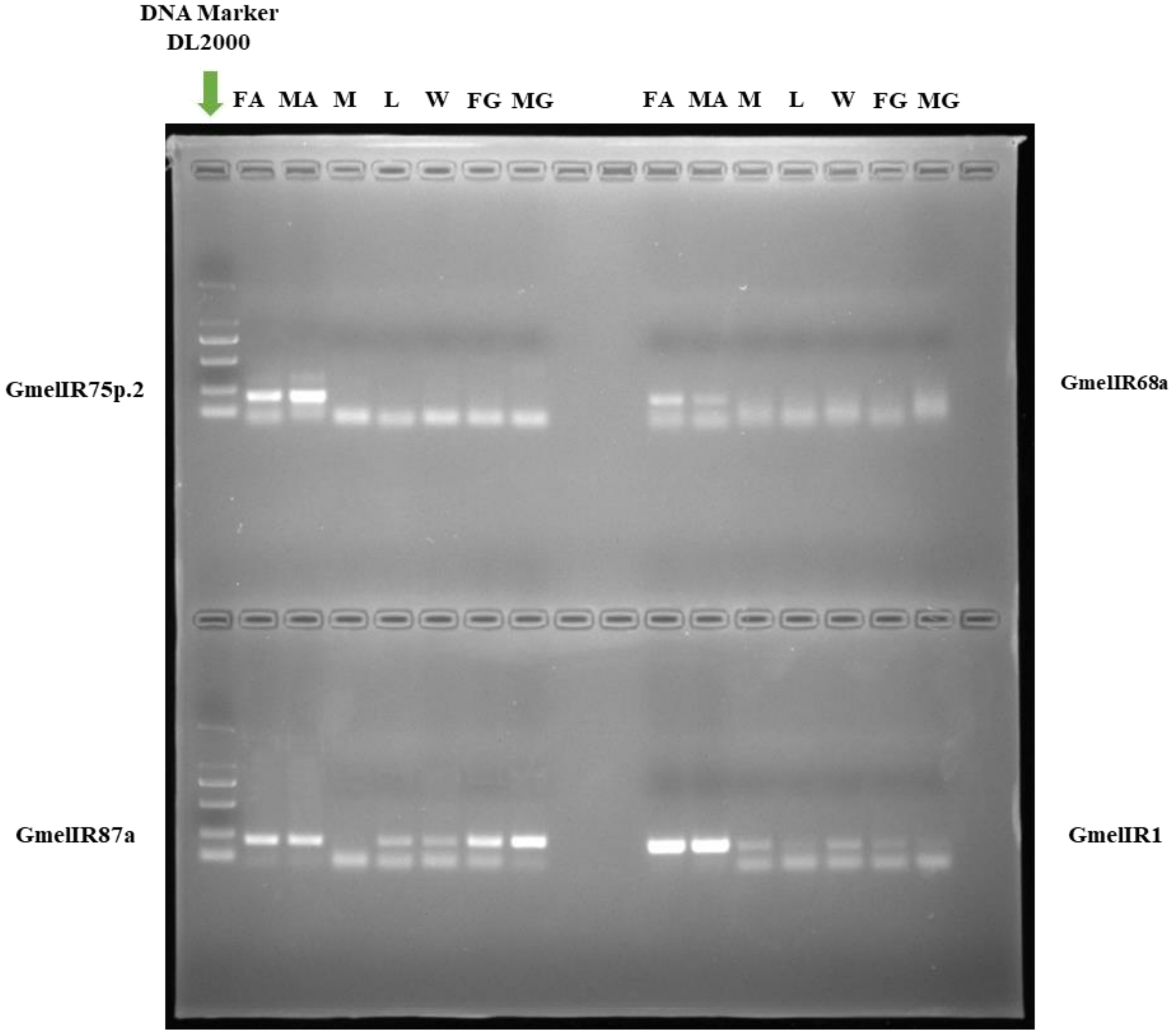

Figure S8. The full-length gel of GmelIR75p.2, GmelIR68a, GmelIR87a and GmelIR1

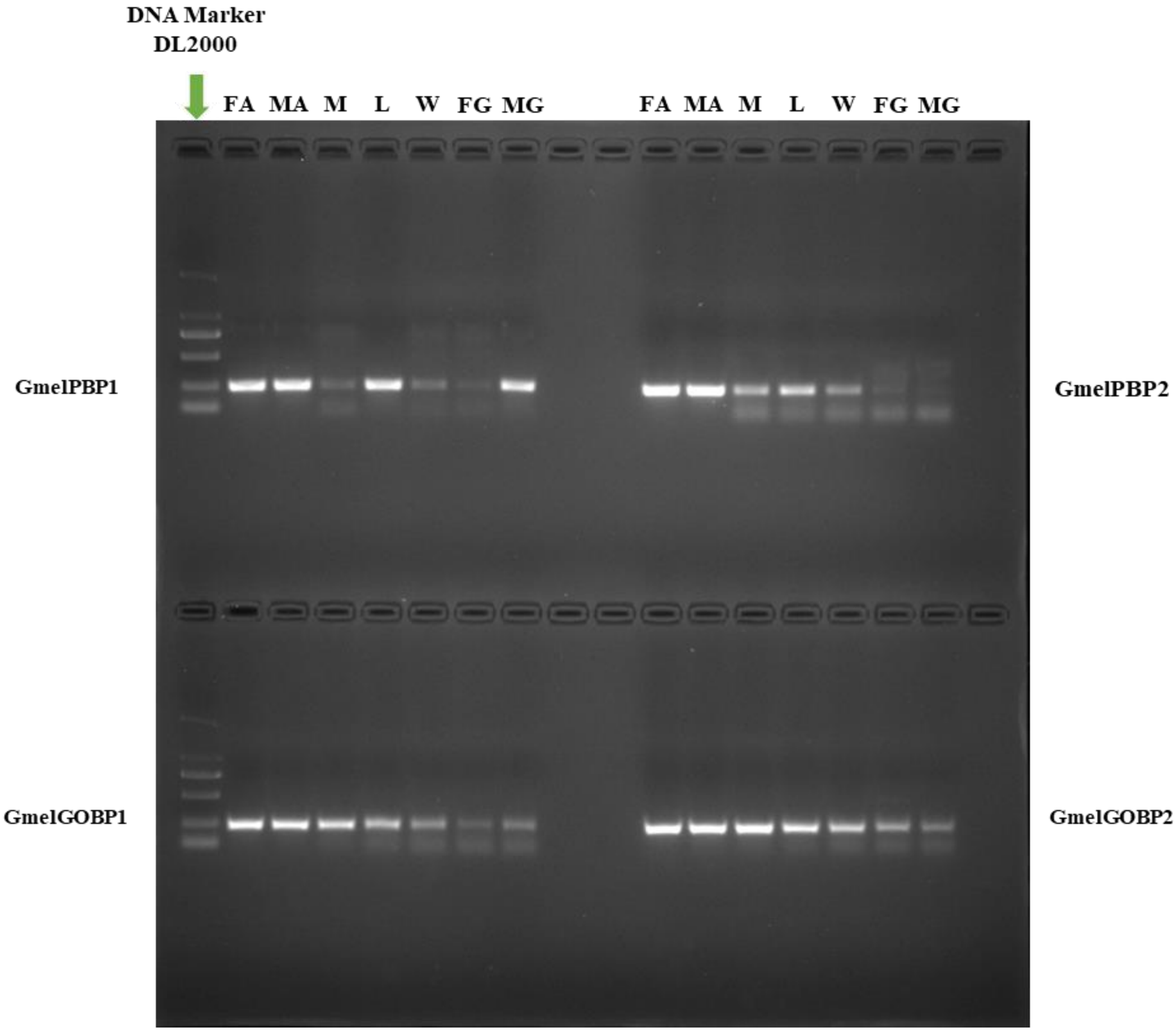

Figure S9. The full-length gel of GmelPBP1, GmelPBP2, GmelGOBP1 and GmelGOBP2

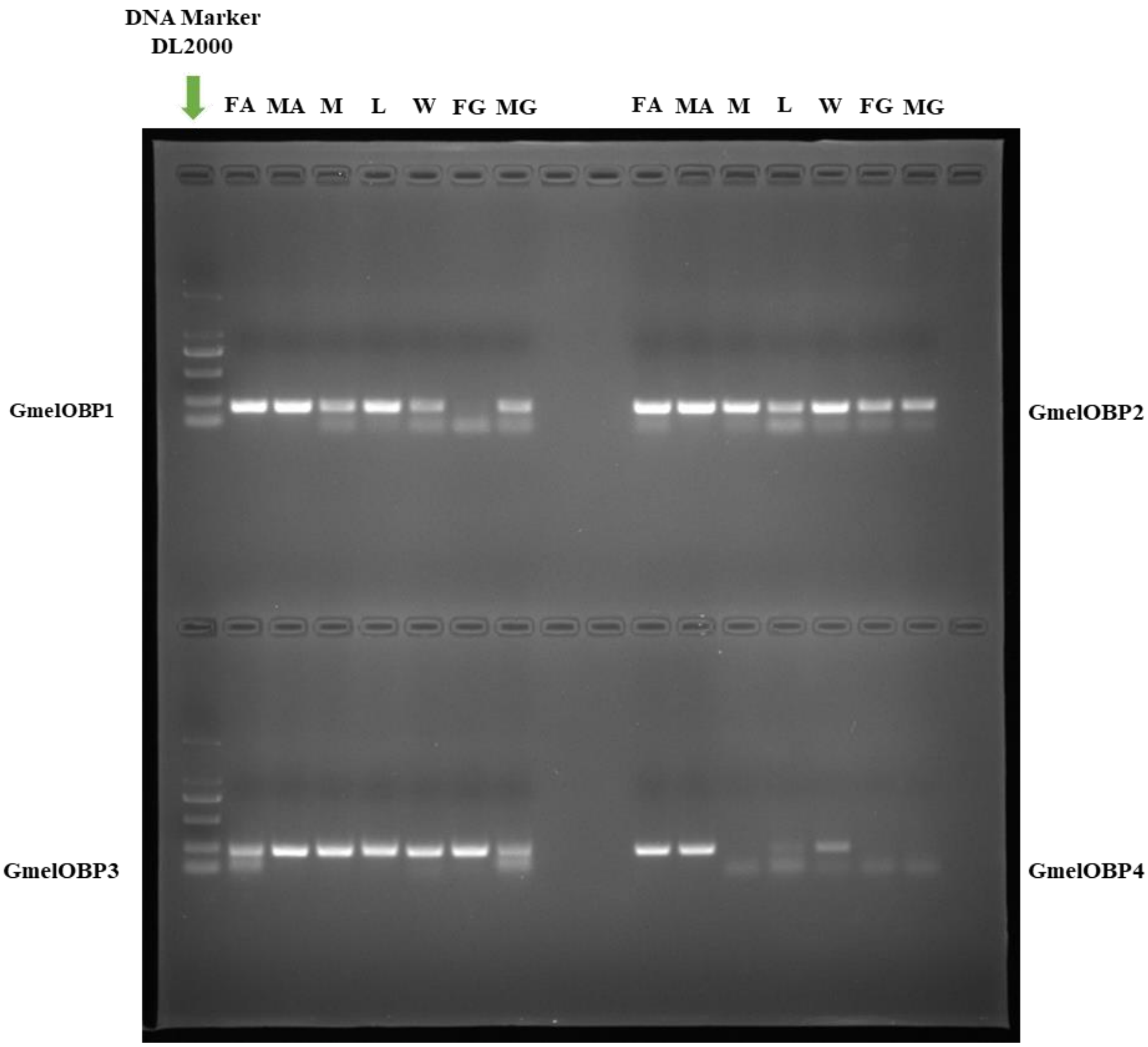

Figure S10. The full-length gel of GmelOBP1-4

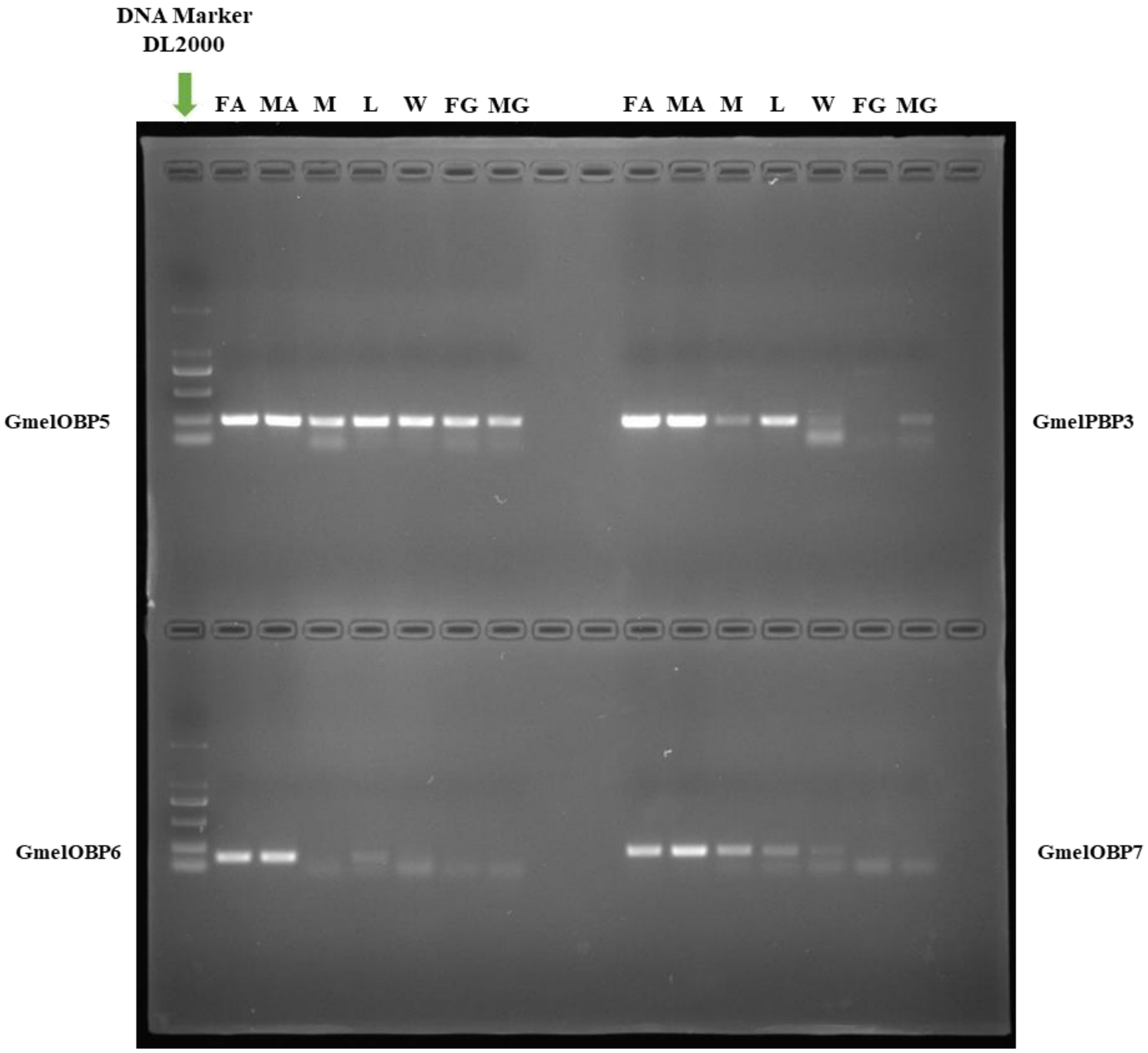

Figure S11. The full-length gel of GmelOBP5, GmelPBP3, GmelOBP6 and GmelOBP7

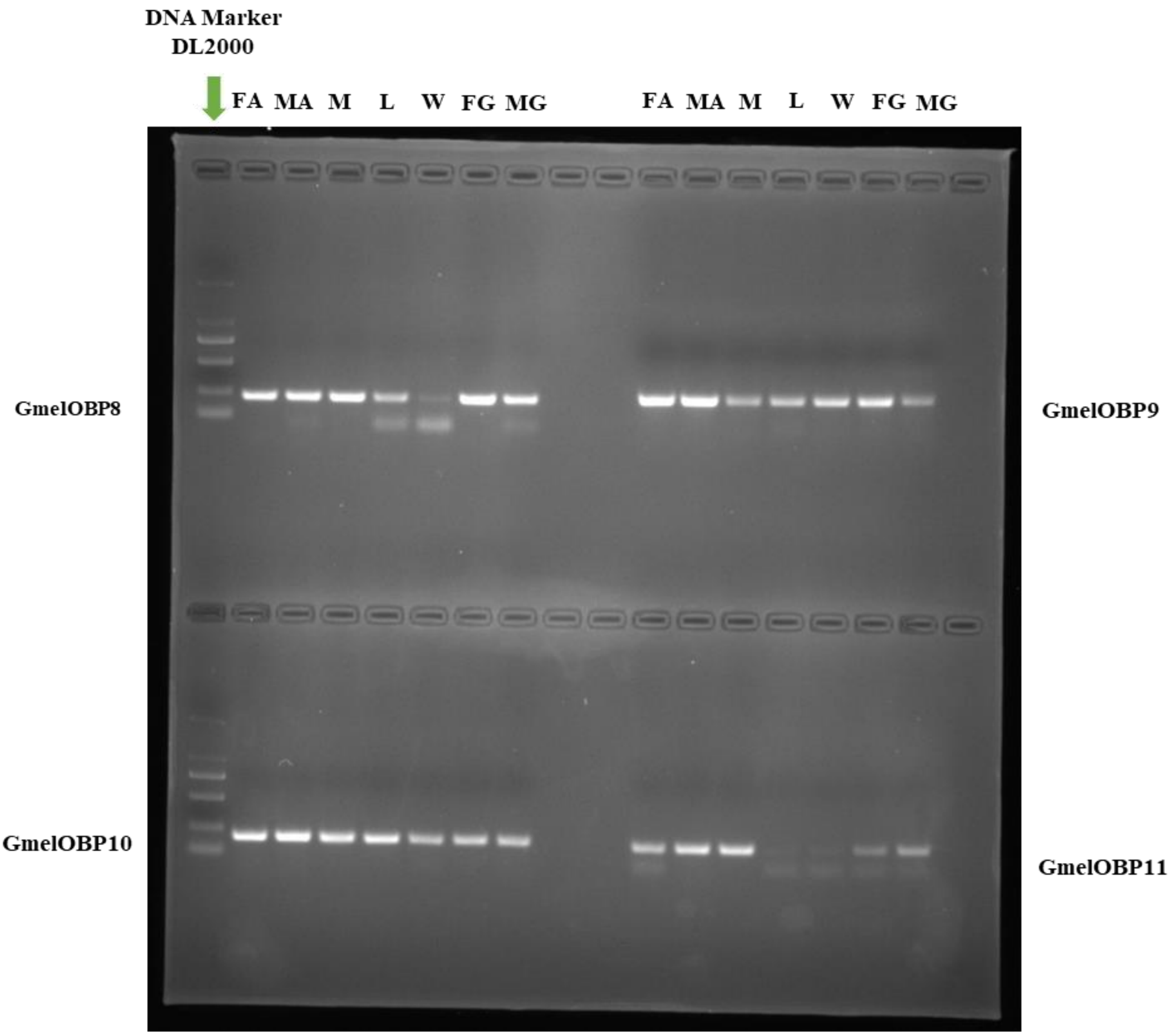

Figure S12. The full-length gel of GmelOBP8-11

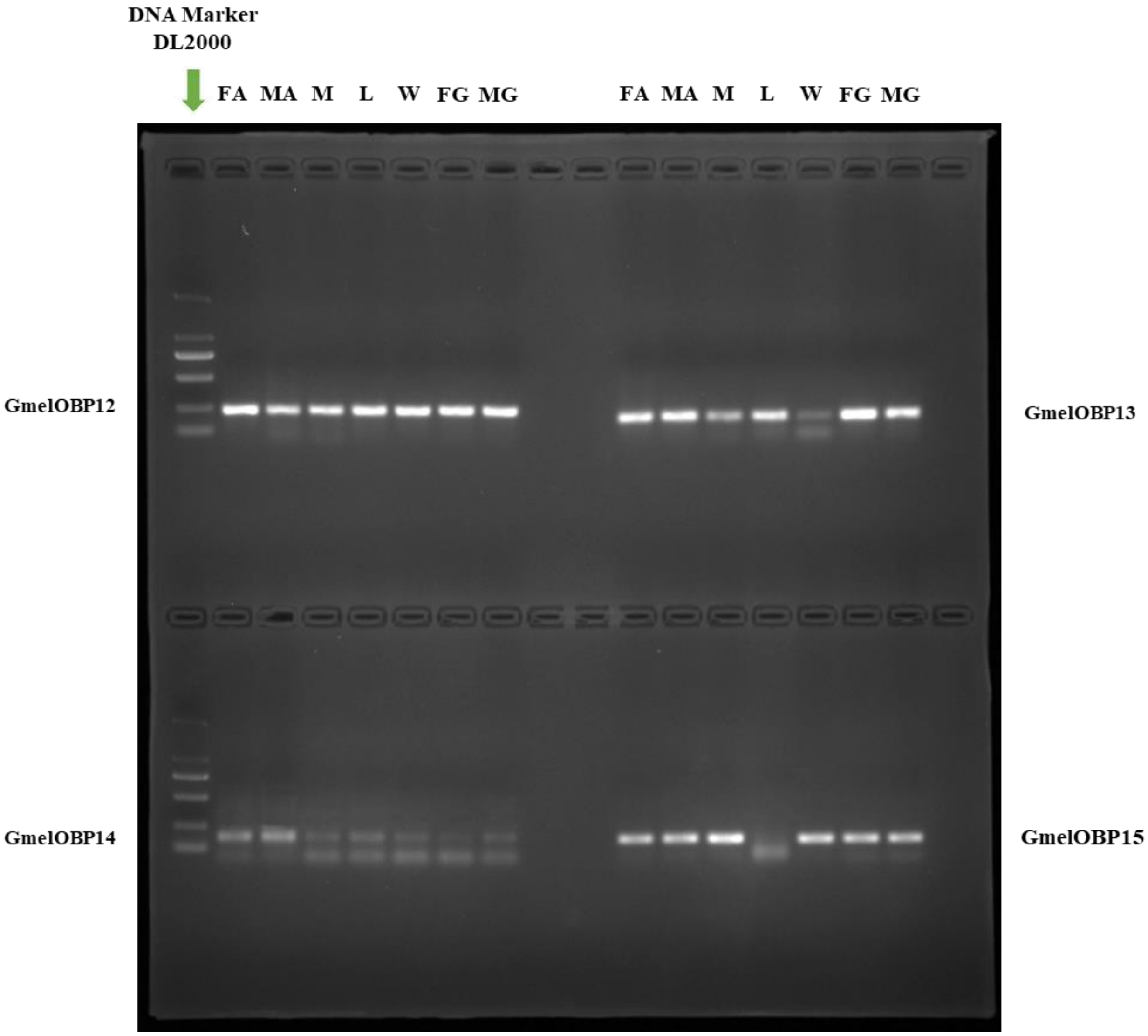

Figure S13. The full-length gel of GmelOBP12-15

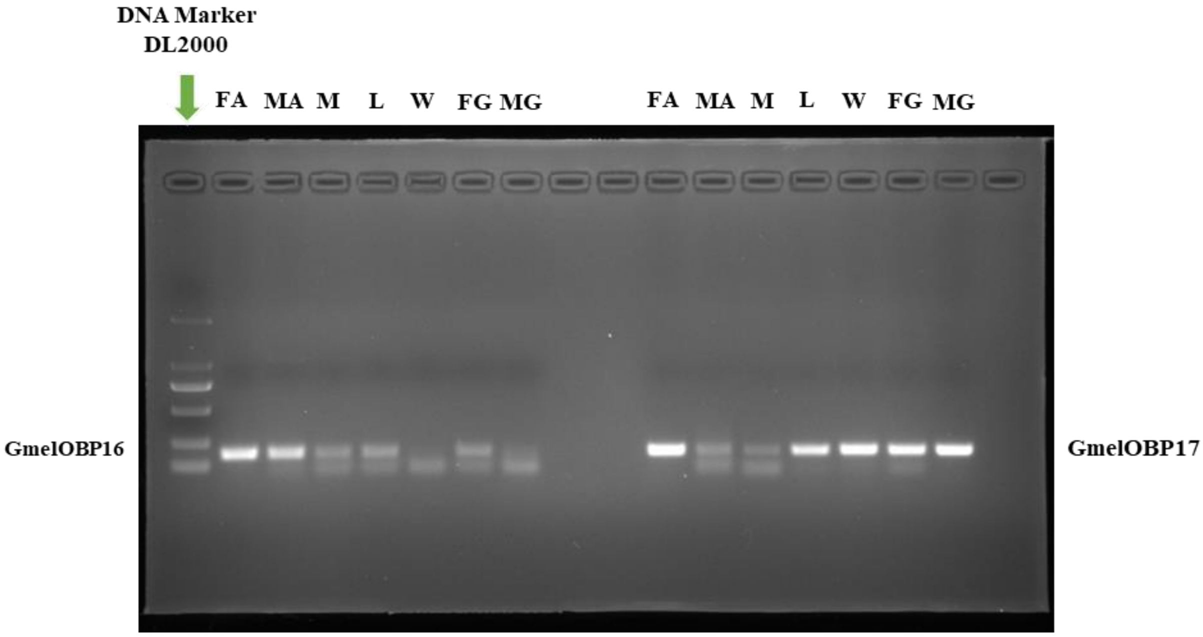

Figure S14. The full-length gel of GmelOBP16-17

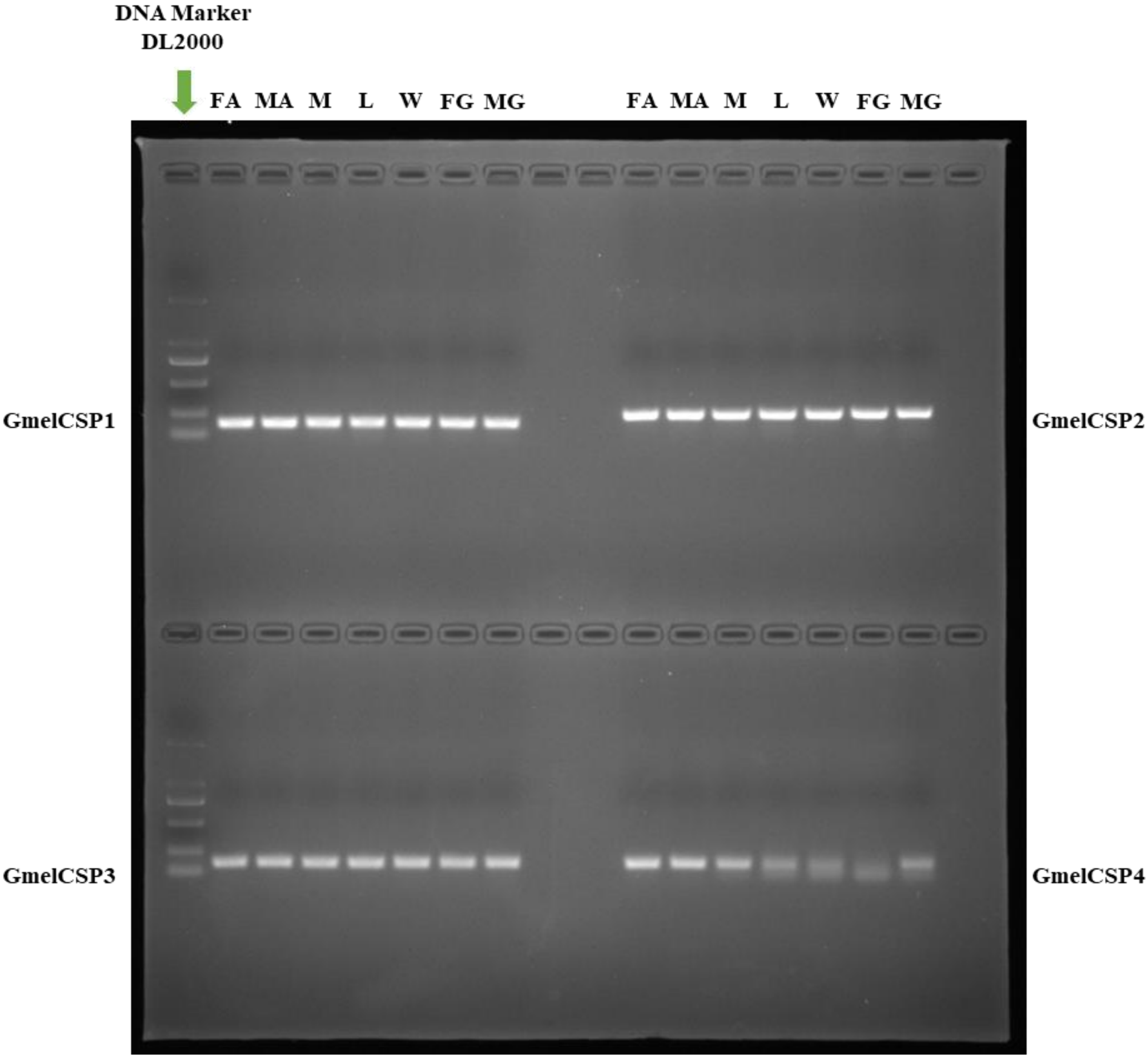

Figure S15. The full-length gel of GmelCSP1-4

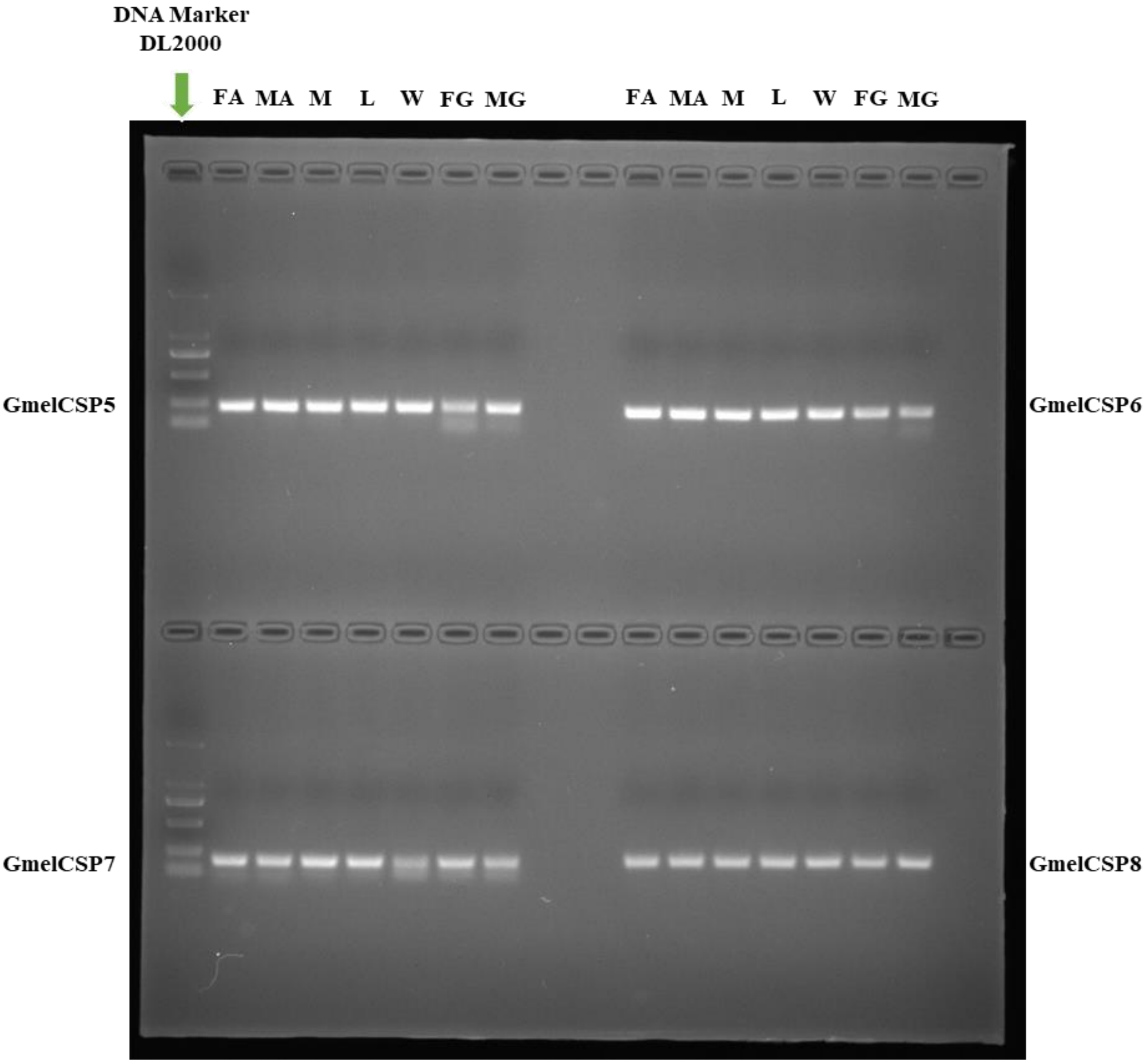

Figure S16. The full-length gel of GmelCSP5-8

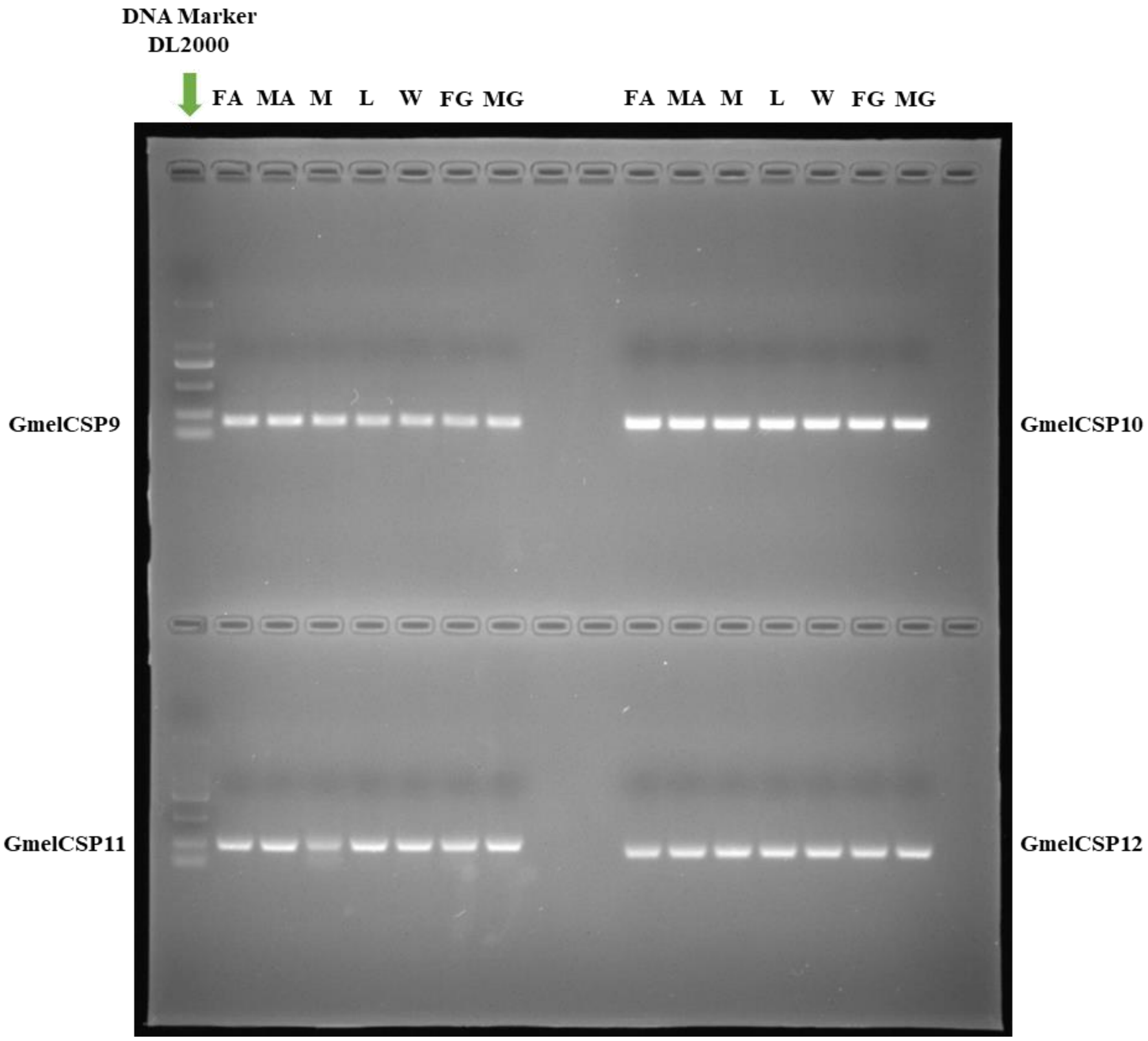

Figure S17. The full-length gel of GmelCSP9-12

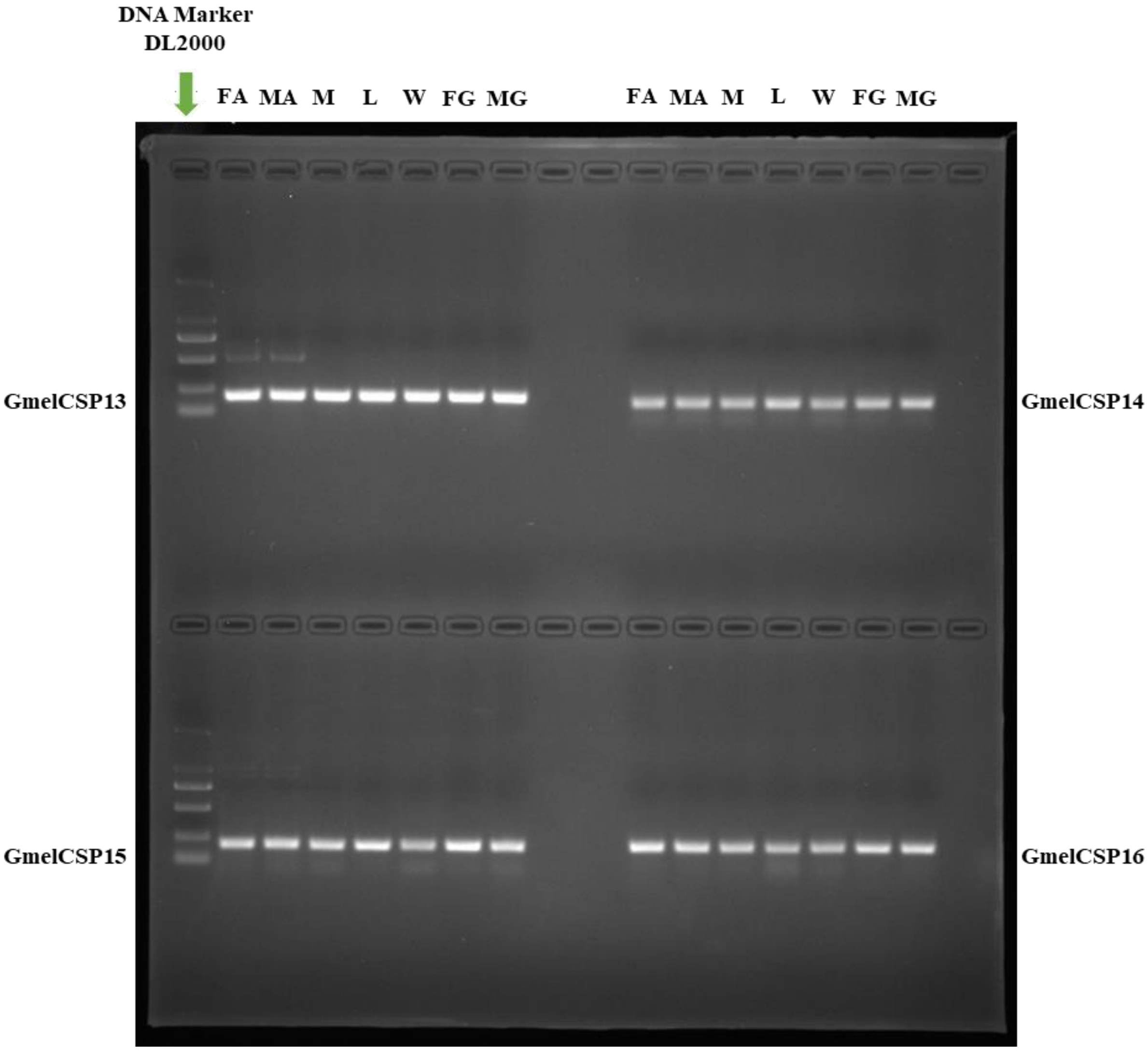

Figure S18. The full-length gel of GmelCSP13-16

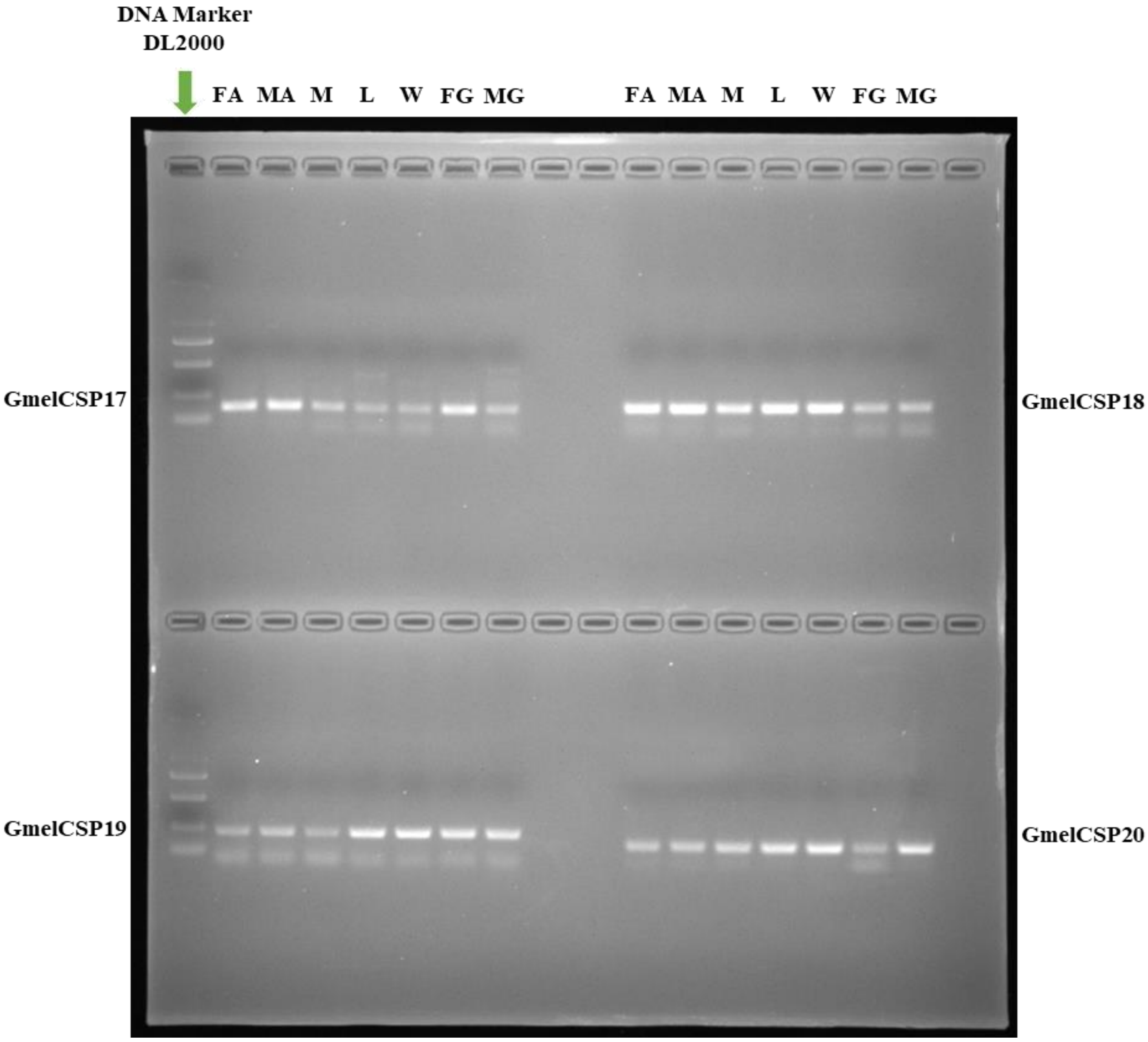

Figure S19. The full-length gel of GmelCSP17-20
